# Biofilm development of an opportunistic model bacterium analysed at high spatiotemporal resolution in the framework of a precise flow cell

**DOI:** 10.1038/npjbiofilms.2016.23

**Published:** 2016-10-19

**Authors:** Chun Ping Lim, Phuong Nguyen Quoc Mai, Dan Roizman Sade, Yee Cheong Lam, Yehuda Cohen

**Affiliations:** 1 Singapore Centre for Environmental Life Sciences Engineering (SCELSE), Nanyang Technological University, Singapore; 2 The School of Mechanical and Aerospace Engineering, Nanyang Technological University, Singapore; 3 The School of Biological Science, Nanyang Technological University, Singapore

## Abstract

Life of bacteria is governed by the physical dimensions of life in microscales, which is dominated by fast diffusion and flow at low Reynolds numbers. Microbial biofilms are structurally and functionally heterogeneous and their development is suggested to be interactively related to their microenvironments. In this study, we were guided by the challenging requirements of precise tools and engineered procedures to achieve reproducible experiments at high spatial and temporal resolutions. Here, we developed a robust precise engineering approach allowing for the quantification of real-time, high-content imaging of biofilm behaviour under well-controlled flow conditions. Through the merging of engineering and microbial ecology, we present a rigorous methodology to quantify biofilm development at resolutions of single micrometre and single minute, using a newly developed flow cell. We designed and fabricated a high-precision flow cell to create defined and reproducible flow conditions. We applied high-content confocal laser scanning microscopy and developed image quantification using a model biofilm of a defined opportunistic strain, *Pseudomonas putida* OUS82. We observed complex patterns in the early events of biofilm formation, which were followed by total dispersal. These patterns were closely related to the flow conditions. These biofilm behavioural phenomena were found to be highly reproducible, despite the heterogeneous nature of biofilm.

## Introduction

Biofilms are inherently heterogeneous communities. Biofilm activities are not the summation of their individual constituents in their planktonic state, but rather, the cells in biofilms exhibit distinctly different properties from their single freely-suspended homogeneous cultures.^1,2^ The close physical proximity among microorganisms enables small molecules to diffuse effectively, allowing for quorum sensing/quenching signals^3,4^ and may allow for the regulation of specific biofilm developmental patterns.^5–8^

The initial phases of biofilm development are tightly correlated to their microenvironments. The importance of environmental gradients in selecting the most suitable microbial physiology to thrive in a given ecological niche was described already in the late 19th century by Sergey Winogradsky.^9^ Quantification of these important observations has since been limited by the lack of tools for studying biofilm development from single cells to social communities. The heterogeneous nature of biofilms requires precise tools and procedures to allow for the quantification of their behaviour at the appropriate resolutions and at low background noise.

The study of microbial biofilms using traditional microbiology methods, like agar plates, microtiter trays, etc. are limited to non-flow environments that biofilms rarely encounter in nature. Ecological interactions are suggested to govern the development of microbial biofilms.^10^ Therefore, biofilms should be studied under conditions that mimic their natural habitats such as biofilm flow cells. This concept led to the design of the presently most widely used growth chamber, with growth media pumped into straight channels by peristaltic pumps. Developed by Wolfaardt *et al.*^11^ and later refined by Christensen *et al.,*^12^ these straight channel platforms provide unidirectional flow fields operated under well-established protocols.^13^ Efforts to increase flow cell sophistication also resulted in a system for generating a two-dimensional flow pattern.^14^ However, the ability to create specific well-controlled environments, including defined gradients, in these chambers remained limited.

The application of various lab-on-chip technologies into biofilm studies gave rise to various microfluidic flow cells with the capabilities of generating well-defined conditions such as chemical gradients,^15–18^ hydrodynamic stresses^19–22^ and temperature gradients.^23^ However, these chambers are often custom-made for specific experiments. Moreover, most microfluidic devices are confined to shallow channels, and do not allow for accumulating sufficient biomass without the biofilm significantly altering the bulk environment even in the initial stages of biofilm development. An additional limitation of existing flow cells is that the channels are often sealed by permanent bonding, preventing the removal of intact surface-associated biofilm for further analyses, usually requiring the substrata to be destructively broken.^13^

Recent developments in fluorescence microscopy, coupled with advances in fluidics and microfabrication facilitate dynamic biofilm studies by real-time live imaging at the spatial and temporal resolutions required to unravel the physics that shapes life at microscales. To achieve this, single micrometre spatial resolution and single minute temporal resolution are necessary.^24,25^ Precision has to be incorporated into a flow cell system from its design, fabrication and the operation protocols. We present here a high spatiotemporal resolution approach for the real-time study of biofilm behaviour under well-controlled flow conditions. This flow cell was fabricated by micro-machining processes that were optimised for precision and reproducibility. In addition, the chamber has a removable substrate that allows for pre-treatments of the surface by surface modifications and for downstream analyses of the intact biofilm developed on that surface.

Using confocal laser scanning microscopy, we demonstrated live imaging at multiple locations in our flow cell over long-term experiments. Each specific position inside the chamber can be revisited at any time during the experiments with an accuracy of ±2 μm limited only by the accuracy of the motorised stage of the microscope. We present a protocol to operate the flow cell including validation of flow pattern, biofilm experiments and quantitative biofilm growth analysis. Using the developed procedures, we demonstrate and quantify for the first time the complex yet highly reproducible dynamic formation and dispersal patterns in a model biofilm using a well-documented strain of *Pseudomonas putida* OUS82.

## Results

The aim of this study was to develop robust procedures to observe the behaviour patterns of the initial phases of biofilm development from attachment to biofilm build-up and dispersal. These observations were conducted under defined flow conditions. By applying a precise engineering approach for an in-depth understanding of life in microscales, we have observed and quantified, for the first time, fundamental developmental features during the initial phases of biofilm formation and dispersal.

We have designed, fabricated and validated a novel biofilm flow cell; assembled a system of precise flow control and mounted this system on an accurate motorised stage of an advanced confocal microscope; acquired high-resolution images and reproducible real-time imaging in three dimensions. The large number of confocal images were then quantified and analysed.

### Hyperbolic biofilm flow cell

**Figure 1:**
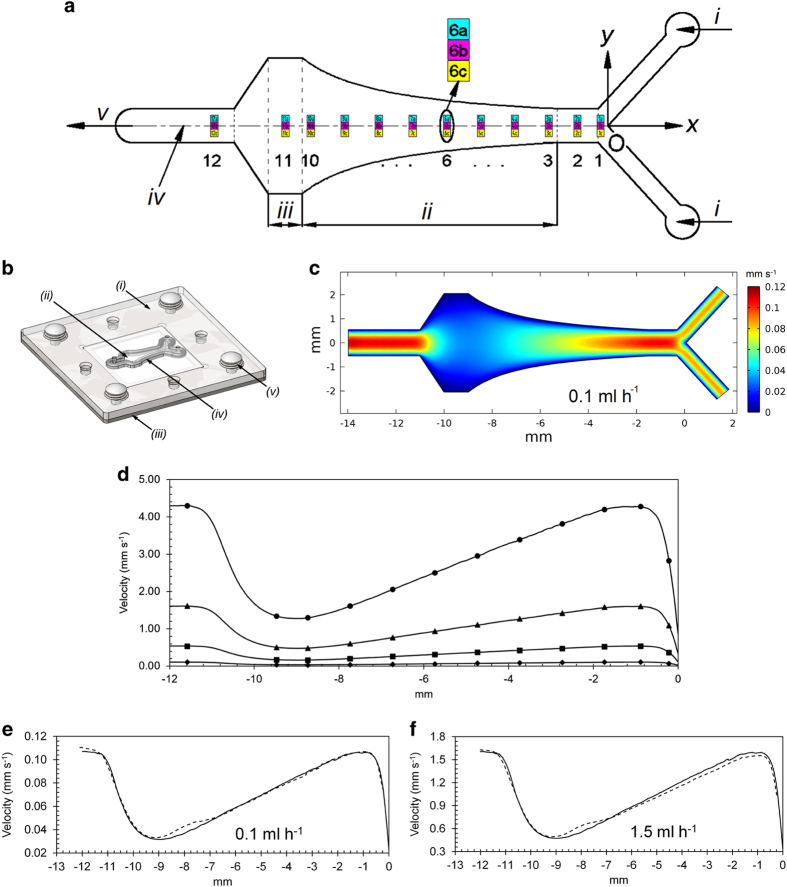
Structure and flow field validation of the flow cell. (**a**) Hyperbolic channel profile with two inlets (i) feeding into a hyperbolic expansion (ii) leading to one outlet (v). The flow direction is always indicated from right to left. The intersection of the two inlets was chosen as the zero reference point (O). Twelve positions of observation were selected along the *x* direction. At each position, adjacent three areas were examined, colour coded as ‘a’ (top, cyan colour), ‘b’ (middle, magenta colour) and ‘c’ (bottom, yellow colour). The *x*-axis includes positions 1 and 2 as controls, positions 3 to 10 covering the hyperbolic expansion (ii). Position 11 is at the exit of the expansion (iii), leading to position 12 as an additional control. The distances between these 36 positions can be found in Supplementary Figure 1b; (iv) is the flow cell centreline. (**b**) The three-dimensional drawing of the flow cell including the channel plate (i) containing the hyperbolic channel (ii), metal backing plate (iii), O-ring seal (iv) and snap-lock pins with compression springs (v). (**c**) Simulated mid-plane flow velocity values (colour coded from 0.00 mm/s to 0.12 mm/s) at flow rate *Q*=0.1 ml h^−1^ per inlet. (**d**) Simulated flow velocity at centre-line for flow rates of 0.1 ml h^−1^ per inlet (◆), 0.5 ml h^−1^ per inlet (■), 1.5 ml h^−1^ per inlet (▲) and 4.0 ml h^−1^ per inlet (●) with the markers indicating the positions of the imaging. (**e** and **f**) Comparison of simulated (continuous line) and measured (dashed line) flow velocity at centre-line at flow rates of (**e**) 0.1 ml h^−1^ per inlet and (**f**) 1.5 ml h^−1^ per inlet. The horizontal axes in graphs **c**–**f** are the *x* coordinates of the flow cell with the origin at O.

The flow cell we developed has a channel with two inlets feeding into a hyperbolic expansion that then leads to one outlet (Figure 1a and Supplementary Figure 1b). The hyperbolic expansion generates a linearly decreasing flow velocity (i.e., a negative linear velocity gradient) along the channel center-line (zone ii in Figure 1a). The flow fields for four flow rates used (*Q*=0.1, 0.5, 1.5 and 4.0 ml h^−1^ per inlet) were first simulated (Figures 1c and d) and then experimentally validated by particle image velocimetry, with good agreement between the simulated and measured flow velocities (Figures 1e and f). The flow velocity decreased linearly from *x*=−1.49 mm to *x*=−8.99 mm resulting in a three times difference in magnitude at these two locations at all flow rates.

### Real-time high-content three-dimensional imaging

We observed the biofilm development of green fluorescent protein (GFP)-tagged *P. putida* OUS82^26^ under defined flow fields using confocal laser scanning microscopy at real time, high content and high resolution. Each experiment, which was conducted for up to 12 h 50 min, generated up to 42,000 images, taken at 36 locations at an interval of 10 min (Supplementary Table 1).

**Figure 2:**
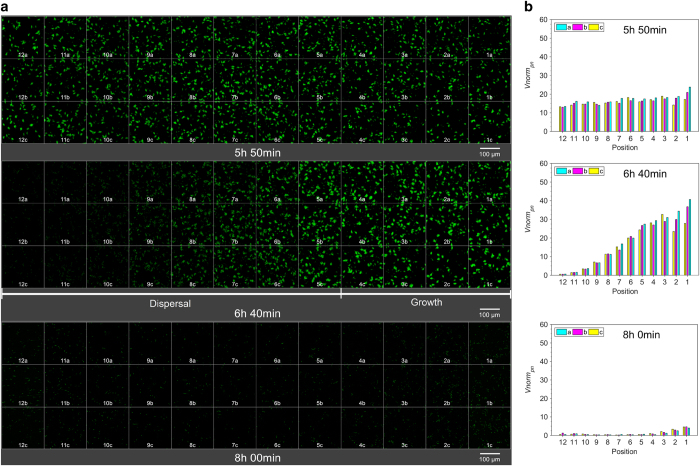
Dynamic nature of *P. putida* biofilm formation and dispersal at low flow rate *Q*=0.1 ml h^−1^ per inlet. (**a**) Confocal microscopy images of *P. putida* OUS82::GFP biofilm at 36 positions at selected significant time points. The image at each time point is a collage of 36 images, each of which is the maximum intensity projection from the Z-stack of the respective location. (Top) The microcolonies at downstream positions 12 a–c (i.e., leftmost images) reached their maximal growth. Individual bacteria began dispersing from the clusters, whereas growth continued at positions 1–11. (Middle) Dispersal propagated from position 12 up to position 5, whereas the microcolonies at positions 4–1 continued growing. (Bottom) Dispersal was evident at all the positions. Most cells were flushed downstream towards the outlet of the flow cell. The complete biofilm behaviour from bacterial attachment, cluster formation and maturation to dispersal over 8 h 20 min is shown in Supplementary Video 1. (**b**) Normalised total biovolume per imaging window, *V*norm*_pn_*, of the biofilm shown in Figure 2a. (Top) Microcolonies at positions 12 a–c reached their highest biovolume corresponding to their maximal growth. Except at locations 1–2, there are small variations in *V*norm*_pn_* at the three positions along the *y* direction for each position along the *x*-axis, showing that biofilm growth is consistent; (Middle) The gradually increasing *V*norm*_pn_* from positions 12 to 1 demonstrates the different stages of microcolony development along the flow direction. (Bottom) *V*norm*_pn_* at all positions drop significantly showing that the cells were flushed out of the flow cell. The complete set of biovolume over the entire 8 h 20 min-period imaging can be found in Supplementary Videos 5 (with normalisation) and 6 (without normalisation). GFP, green fluorescent protein.

**Figure 3:**
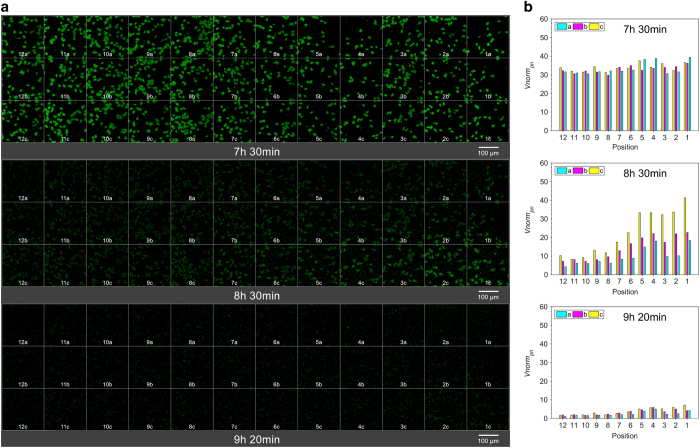
Dynamic nature of *P. putida* biofilm formation and dispersal at high flow rate *Q*=1.5 ml h^−1^ per inlet. (**a**) Confocal microscopy images of *P. putida* OUS82::GFP biofilm at 36 positions at selected significant time points. (Top) Microcolonies observed at the downstream section of the flow cell (positions 12 a–c, leftmost images) reached their maximal growth. Concomitantly, the initiation of dispersal at the same location was observed. (Middle) Dispersal initiated at downstream and then propagated upstream at a high rate. (Bottom) Dispersal happened at all the positions. The images displayed at each position in each time point is the maximum intensity projection of the Z-stacks. The complete development behaviour covering 10 h 20 min is shown in Supplementary Video 3. (**b**) Normalised total biovolume per imaging window, *V*norm*_pn_*, of the biofilm shown in Figure 3a. (Top) Microcolonies at positions 12 a–c reached their highest biovolume. *V*norm*_pn_* at the three adjacent positions along the *y* direction showed little variation, indicating consistent biofilm growth. (Middle) *V*norm*_pn_* at all the positions drops, indicating dispersal. (Bottom) *V*norm*_pn_* at all the positions reduces significantly as the cells were flushed away towards the outlet. The complete data of 10 h 20 min-period development behaviour is shown in Supplementary Videos 5 (with normalisation) and 6 (without normalisation). GFP, green fluorescent protein.

The images were compiled into snapshots (Figures 2a and 3a) and four videos (Supplementary Videos 1–4), revealing the dynamic nature of biofilm formation and dispersal in this model organism. In all the experiments, we observed gradual biofilm formation up to a maximal biovolume followed by total dispersal. Dispersal was always observed to initiate at downstream (position 12) and propagate upstream (position 1). Significantly, different dynamics of biofilm formation and dispersal patterns were observed when comparing the results obtained under the four flow rates used. At the lowest flow rate of 0.1 ml h^−1^, maximal biovolume was observed at 5 h 57 min (Figure 2a—top) followed by the onset of a dispersal event, which then further propagated upstream (Supplementary Video 1) and full dispersal was observed at 8 h (Figure 2a—bottom). Under a higher flow rate of 1.5 ml h^−1^, the onset of the dispersal process was delayed by 1 h 41 min (Figure 3a and Supplementary Video 3). In addition, larger micro-aggregates were observed under a high flow rate just before the initiation of the dispersal. In these low and high flow rates, the first dispersal was observed at the same downstream positions of 12 a–c. Yet the propagation of dispersal towards upstream was much faster at the higher flow rate. Under the higher flow, initiation of dispersal propagated to the entire chamber within 32 min, whereas it took 63 min at the low flow rate. Similarly, fast dispersal propagation was also observed when examined under additional flow rates of 0.5 ml h^−1^ (Supplementary Video 2) and 4.0 ml h^−1^ (Supplementary Video 4). Repeated experiments clearly demonstrated the reproducibility of these patterns. The same behaviours were observed to be repeatable within temporal resolutions of 4–17 min (Table 1).

The large number of collected microscopic images were analysed to quantify the dynamics of the biofilm behaviour (Figures 2b and 3b, Supplementary Videos 5 and 6). The propagation of dispersal under the low flow rate of 0.1 ml h^−1^ was gradual, starting at the downstream position 12 while biofilm development was still obvious at the upstream positions (Figure 2b—middle). Dispersal was completed in the entire chamber during the following 1 h 20 min. The onset of dispersal was delayed and the duration for total dispersal was shortened down to 50 min under 1.5 ml h^−1^ (Figure 3b and Supplementary Video 5). Under the highest flow of 4.0 ml h^−1^, we observed a further delay in the onset of dispersal to 9 h (Supplementary Video 5). The propagation was abrupt, too fast to be accurately defined given the temporal resolution of 10 min used in this research.

**Figure 4:**
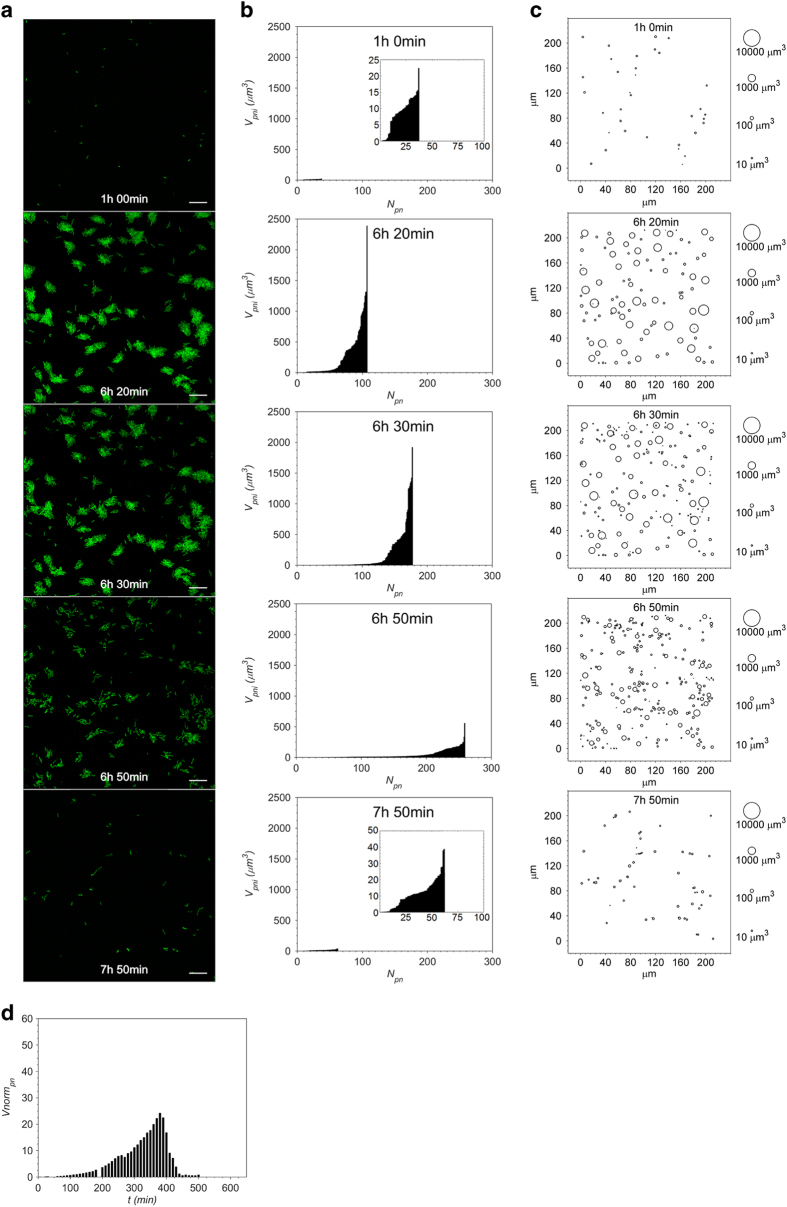
Dynamics of *P. putida* OUS82::GFP clusters formation and dispersal at position 7a at significant time points under low flow rate *Q*=0.1 ml h^−1^ per inlet. (**a**) Confocal images over the time course of initial biofilm development—from attachment to dispersal: 1 h (initial attached bacteria), 6 h 20 min (maximal growth of biofilm cluster before start of dispersal), 6 h 30 min (commencement of dispersal), 6 h 50 min (dispersal of biofilm) and 7 h 50 min (fully dispersed biofilm, some *P. putida* cells that remained on the surface resembled filaments). Scale bar: 20 μm (applied to Figure 4a). Supplementary Video 7 shows the 8 h 20 min-period time lapse of the biofilm development at this position. A three-dimensional view and a cross-section view of the confocal image at 6 h 20 min are shown in Supplementary Figures 3a and b, respectively. (**b**) The distribution of cluster size corresponding to the biofilm images in **a**. The *y*-axis is the individual cluster biovolume, *V_pni_*, while the *x-*axis is the total number of clusters present in the imaging window, *N_pn_*. Supplementary Video 8a shows the changes in cluster size distribution over the entire imaging period at this position. (**c**) Bubble plot of the spatial distribution of *V_pni_* for the corresponding time point in **a**. Supplementary Video 9 shows the bubble plot corresponding to the biofilm development at position 7a over 8 h 20 min. (**d**) Normalised total biovolume, *V*norm*_pn_*, at position 7a over time, *t*. The defined time of the initiation of dispersal is identified from the decrease in *V*norm*_pn_* (immediately after the highest peak). Supplementary Video 10 shows total biovolume per imaging window, *V_pn_*, versus *t* for 36 positions. GFP, green fluorescent protein.

Biofilm images taken at one selected position (7a), along with the quantification of these images (Figure 4, Supplementary Figures 2 and 3 and Supplementary Video 7) demonstrate the different kinetics of biofilm formation and dispersal patterns in correlation to the four flow rates. Dispersal by sloughing was observed only when the biofilm was exposed to the highest flow rate of 4.0 ml h^−1^ (Supplementary Video 11).

At this position under the lowest flow rate (Figure 4a), the following sequence of events was apparent: bacteria attachment; development into maximal cluster size; initiation of dispersal; biofilm clusters decreased in size and gradually broke up; and followed by full dispersal. Eventually, only a small number of individual bacteria remained adhered to the surface.

The distribution of cluster size for the lowest flow (Figure 4b) was significantly different from that of the higher flows (Supplementary Figure 2b and Supplementary Videos 8a–b), and the bubble plots of the clusters’ biovolume shown in Figure 4c and Supplementary Video 9, enabled further quantification of these processes. One hour into the experiment, the attached bacteria were arranged as single cells and small clusters with biovolume of less than 25 μm^3^. The biofilm reached its maximal biovolume at time 6 h 20 min. At this time, the largest cluster reached a maximum biovolume of 2,388 μm^3^. At 6 h 30 min, a surge in the total number of clusters was observed as indicated by the shift of the clusters distribution to the right. The concomitant drop in the clusters biovolume was the expression of the commencement of dispersal. These events were further enhanced during the next 30 min, corresponding to an additional increase in the number of new smaller clusters, which implied clusters breakup and the drifting of newly dispersed cells from the upstream locations.

The onset of dispersal was delayed with increasing flow rate, from 6 h 34 min at 0.1 ml h^−1^ to 9 h 5 min at 4.0 ml h^−1^. Concomitantly, the observed biovolume of the biggest cluster increased from 2,388 μm^3^ at 0.1 ml h^−1^ to 29,535 μm^3^ at 4.0 ml h^−1^. This phenomenon was observed along with the narrowing in cluster size distribution with increasing flow rates.

When analysing the spatial distribution of the clusters (Figure 4c and Supplementary Video 9), we followed the changes in the following parameters: increase of each aggregate; merging of spatially close aggregates and the addition of newly emerging clusters. The normalised total biovolume per area, *V*norm*_pn_*, at this position was plotted over time (Figure 4d) to calculate the communal doubling time in addition to determining the time at maximal biovolume and the commencement of dispersal. Total biovolume over time for the other positions is shown in Supplementary Video 10.

**Figure 5:**
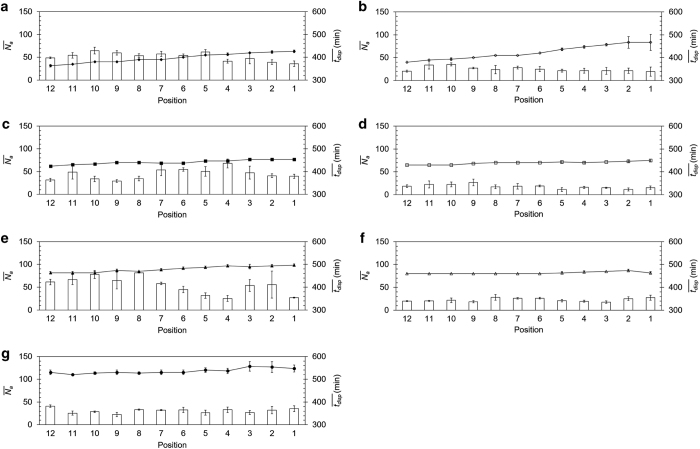
Average number of initially attached cluster, $\overset{¯}{N_{a}}$, (bar chart) and the time at the initiation of biofilm dispersal, $\overset{¯}{t_{disp}}$, (line) at 12 positions for (**a**) 0.1 ml h^−1^ (Run 1—◆) (**b**) 0.1 ml h^−1^ (Run 2—◊) (**c**) 0.5 ml h^−1^ (Run 1—■) (**d**) 0.5 ml h^−1^ (Run 2—□) (**e**) 1.5 ml h^−1^ (Run 1—▲) (**f**) 1.5 ml h^−1^ (Run 2—Δ) (**g**) 4.0 ml h^−1^ (Run 1—●). $\overset{¯}{N_{a}}$ is the number of clusters attached (at *t*=2 h) averaged over **a**–**c** positions of the respective locations along the *x* axis. $\overset{¯}{t_{disp}}$ is the average (over positions **a**–**c**) of the time at initiation of dispersal at the respective locations along the *x* axis.

**Figure 6:**
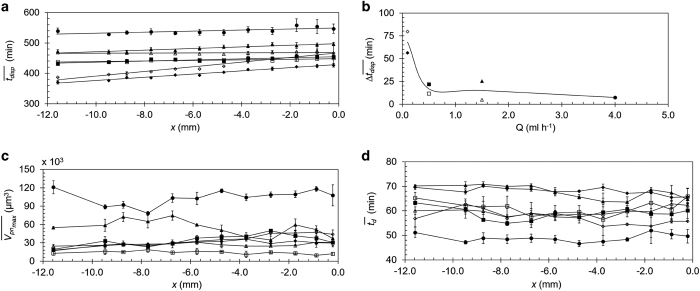
Biofilm development and dispersal kinetics in relation to the positions in the flow cell. (**a**) Time at the initiation of dispersal, $\overset{¯}{t_{disp}}$, (averaged over **a**–**c** positions at each *x* location) at the 12 measured positions along the flow cell. (**b**) The time difference of dispersal initiation between downstream (position 12) and upstream (position 1), $\Delta \overset{¯}{t_{disp}}$, for four flow rates. (**c**) The maximal biovolume at the 12 positions, $\overset{¯}{V_{pn_{\max}}}$, (averaged over **a**–**c** positions) before dispersal. (**d**) The doubling time, $\overset{¯}{t_{d}}$, (averaged over **a**–**c** positions) at the 12 positions. The flow rates and their corresponding symbols are: ◆ 0.1 ml h^−1^ (Run 1), ◊ 0.1 ml h^−1^ (Run 2), ■ 0.5 ml h^−1^ (Run 1), □ 0.5 ml h^−1^ (Run 2), ▲ 1.5 ml h^−1^ (Run 1), Δ 1.5 ml h^−1^ (Run 2), ● 4.0 ml h^−1^ (Run 1).

Figure 5 defines the level of reproducibility achieved under the experimental procedures established in this study. It shows the timing of the initiation of dispersal at each position at the various flow rates examined in this study and compared with the average number of initially attached clusters in each of these positions $\left(\overset{¯}{N_{a}}\right)$. Reproducibility is defined by (a)—the variations among the three adjacent areas for each position along the *x*-axis at each time point (error bars in Figures 5 and 6 are standard deviations); and (b)—repeating the entire experiment for three different flow rates of 0.1 ml h^−1^, 0.5 ml h^−1^ and 1.5 ml h^−1^ (Figures 5a–f).

Although the average number of initially attached clusters shown in bars varies significantly, it has little effect on the trend of initiation of dispersal shown in the line.

Figures 6a–d examine the biofilm behaviour at all the positions in all the experiments in an attempt to quantify the upstream–downstream relation of the kinetics of biofilm formation and dispersal: (a)—average initial dispersal time $\left(\overset{¯}{t_{disp}}\right)$; (b)—difference between upstream–downstream dispersal time $\left(\Delta \overset{¯}{t_{disp}}\right)$; (c)—average maximal biovolume per imaging area $\left(\overset{¯}{V_{pn_{\max}}}\right)$; and (d)—average calculated doubling time $\left(\overset{¯}{t_{d}}\right)$. Comparing the lines in Figure 6a, the delay in the initiation of dispersal with increasing flow rates is apparent. Furthermore, the slope of the lines indicates a shift from gradual propagation of dispersal in the lowest flow rate into abrupt dispersal in all positions under higher flow rates. The shift from the gradually propagating dispersal to an abrupt process is further emphasised in Figure 6b. No correlation between flow rate and the resulting maximal biovolume at each position is illustrated in Figure 6c, except for the highest flow rate of 4.0 ml h^−1^, which has a significantly higher maximal biovolume in all positions. In addition, the average doubling time at this flow rate is significantly shorter, down to 47 min, in comparison with the longer doubling times of up to 71 min observed at the lower flow rates.

## Discussion

The application of the precise flow cell and robust procedures for high-resolution observation of biofilm development allows us to revisit the roles of microbial ecology in the development of biofilms. Even when using the presented robust procedures, the ability to reproducibly observe biofilm development in controlled environments is limited to its initial stages.

The mechanism of bacterial adhesion has been well established by K. Marshall.^27^ The initial phases of bacterial attachment were later shown to be guided by niche selection.^28,29^ In this study, we observed the transition from fluctuating numbers of adhered cells to stable, permanently adhered cells following 30 min of static inoculation and 1 h 30 min of flow. In each experiment, the permanently adhered cells then multiplied in a clonal mode at a constant doubling time (averaged over 12 positions) along the entire linear velocity gradient regardless of the significantly different flow along these positions (Supplementary Video 8b). The average doubling time ranged from 49±2 min at the highest flow rate to 68±2 min at the lowest flow rate.

Subsequently, deviation from the theoretical clonal cluster distribution was observed as many single bacteria detached from the concomitantly growing clusters. The newly attached cells and the established clusters continued to grow in size. During this period, there was an increase in the maximal observed biovolume per area from 24,067 μm^3^ at the low flow rate of 0.5 ml h^−1^ to 104,090 μm^3^ under 4.0 ml h^−1^. This significant increase in the maximal biovolume could have been dependent on the overall flux of nutrient supply. Yet, we could not detect significant differences in biovolume (Figure 6c) and doubling time (Figure 6d) along the *x*-axis in each of the experiments albeit having a linearly decreasing nutrient flux as generated by the velocity gradient. This phenomenon repeated itself in all the experiments regardless of the flow rate. Furthermore, there were no significant differences in the biovolumes and doubling times between the two control positions—2 and 12. These two positions have identical velocity in each experiment but are at opposite ends of the channel—position 2 near the medium inflow while position 12 near the medium outflow (Figures 6c and d). These clearly indicate that nutrient flux along the flow cell is not the limiting growth factor.

Only when maximal biovolume was reached, was the initiation of dispersal observed in all the experiments. Dispersal processes are clearly affected by flow and they are delayed with increasing flow rates. Dispersal always began at the downstream region close to the outlet of the flow cell and propagated upstream towards the inlet of the medium inflow. This phenomenon was most pronounced at the lowest flow rate. We postulate that it was affected by metabolites produced at the upstream region affecting the initiation of dispersal downstream. With increasing flow rate, the dilution of metabolites reduced this phenomena significantly.^30^

In all the experiments conducted here, we have observed total dispersal. The possible re-establishment of the biofilm following a total dispersal event is unclear.

The experimental approach presented here is a result of true merging of microbial ecology criteria and engineering at the required precision. The combination of precise tools and robust experimental procedures provide the required reproducibility and high precision to address questions on the importance of microscale heterogeneity during the initial development of biofilm aggregates from a single attached cell to maximal biovolume and subsequent dispersal. We have provided here tools and protocols to study biofilms at the spatial and temporal scales required to quantify the physics of life at microscales. This may well be an effective experimental approach for the microbial ecology study of the inherently heterogeneous biofilm mode of life either in free biofilms or host-associated microbiomes.^31^

## Materials and Methods

### Set-up of flow cell system

Our flow cell system includes (i) syringe pump(s) to precisely regulate flow of media into the flow cell; (ii) tubing to connect different components of the system; (iii) microvalves for flexible media control; (iv) a newly developed hyperbolic flow cell with a removable coverslip; and (v) effluent collectors.

We designed and fabricated a precise flow cell using in-house machining facilities to (a) create controllable well-defined environmental gradients inside the flow cell; (b) have a removable substratum (a microscopy coverslip) on which biofilms develop; this allows for various surface modification and facilitates post-analysis of the intact biofilm developed on the substratum; (c) enable long-term live imaging at multiple positions at high spatial and temporal resolutions. A detailed description of the flow cell structure and performance can be found in our patent.^32^ For this investigation, the flow cell was composed of one disposable acrylic (poly(methyl methacrylate), DAMA Trading, Singapore) plate carrying a channel with a hyperbolic expansion, covered with a removable 22 mm×22 mm×0.17 mm microscopy glass coverslip (Marienfeld Superior, Lauda-Königshofen, Germany). The top view of the channel design and the structure of the flow cell are shown in Figures 1a and b. The channel depth was 0.98 mm for all the experiments and can be easily modified for specific usage. Polymeric components of the flow cell, such as the acrylic plate carrying the channel profile, are designed to be disposable and can be mass produced by injection moulding; therefore lowering its cost, which will aid in the wide adoption of this system.

A two-syringe infusion pump (KDS200, KD Scientific, Holliston, MA, USA) was used to simultaneously control the media flow entering the two inlets of the flow cell. Three-way microvalves (VICI Micro Valve three-port 90° flow path, model JR-660310, Valco Instruments, Houston, TX, USA) were used to provide flexible control of flow during various steps of the experimental process, including removal of air bubbles arising from changing/replenishing media infused into the flow cell. Microbore PTFE Tubing (0.032″ ID×0.056″ OD, Cole-Parmer Instrument Company, Vernon Hills, IL, USA) was used to allow for high temperature resistance, chemical inertness, low gas permeability and a low friction coefficient.

The compact set-up of the flow cell system on a confocal microscope Zeiss LSM 780 (Carl Zeiss, Jena, Germany) for live imaging of biofilm development is visualized in Supplementary Figure 1a. The assembly of the flow cell system is described in detail in Supplementary Note 1.

### Simulation and validation of flow field

We used four flow rates in this study including *Q*=0.1 ml h^−1^ per inlet, *Q*=0.5 ml h^−1^ per inlet, *Q*=1.5 ml h^−1^ per inlet and *Q*=4.0 ml h^−1^ per inlet. We designed and constructed the channel geometry using SolidWorks (Dassault Systèmes SolidWorks, Waltham, MA, USA). Flow fields at all flow rates were simulated using COMSOL Multiphysics 4.2a-Laminar Flow module (COMSOL, Burlington, MA, USA). M9 minimal growth medium supplemented with cassamino acids^33^ (Supplementary Table 2) was used for both the simulation of the flow field and biofilm development experiments. The density and viscosity of the M9 medium required for the simulation were measured to be 1,016±2 kg/m^3^ and 1.09±0.01 mPa s, respectively. For the four flow field simulations, identical flow rates of 0.1 ml h^−1^, 0.5 ml h^−1^, 1.5 ml h^−1^ and 4.0 ml h^−1^ were set at the two inlets and the single outlet was set at atmospheric pressure. No-slip boundary conditions (velocity=0) were imposed on the walls of the channel. The channel was meshed with physics-controlled mesh (calibrated for fluid dynamics) with maximum and minimum element sizes of 300 and 15 μm, respectively. The simulated mid-plane velocity fields (velocity field at half-depth of the channel) and centreline velocities were plotted and subsequently validated experimentally by particle image velocimetry (see Supplementary Note 2).

### Microbial experimental procedure and confocal imaging

A rigorous experimental procedure, including sterilisation and priming ahead of bacterial inoculation were applied to ensure a contaminant-free chamber (Supplementary Figure 4a). A scheme of the flow cell system set-up including two modes of operation is given in Supplementary Figures 4b and c. The flow mode was used as the default setting throughout the experimental procedure, except for a switch to the locked mode during media changing to prevent air being trapped, and during non-flow inoculation.

At the start of each experiment, the flow cell was first sterilised with 70% v/v ethanol (Merck, Singapore) in DDW for 15 min at a flow rate of 1.0 ml h^−1^ per inlet. This was followed by priming the chamber with M9 medium for 15 min at the same flow rate. This step ensured that all ethanol was flushed out. In the next step, the flow cell was inoculated with a suspension of defined strain of *P. putida* OUS82::GFP^26^ that expresses GFP constitutively. This strain was grown overnight in M9 medium and diluted to an optical density of 0.005 at 600 nm and the cell density was measured using colony-forming unit counts (3.43×10^6^±5.51×10^5^ per ml). The inoculum was delivered using an initial flow rate of 4.0 ml h^−1^ per inlet for 1 min to completely fill the tubing from the valves to the two inlets. The flow rate was then reduced to 1 ml h^−1^ per inlet for 4 min. After this stage, the valves were switched to the locked mode for static inoculation. This event was defined as the reference zero time point *t*=0 in all the experiments. Two syringes filled up with M9 media were precisely positioned onto the syringe pump. Any air trapped in the tubing was removed into the two effluent collectors (Supplementary Figure 4c). After 30 min (*t*=30 min), the valves were switched back to the flow mode to allow M9 media flow into the flow cell. This event marked the onset of biofilm development in each experiment. The experimental flow rate for each inlet was set at one of the four flow rates 0.1 ml h^−1^, 0.5 ml h^−1^, 1.5 ml h^−1^ and 4.0 ml h^−1^. All the experiments were conducted at 24 °C.

Microscopic imaging of the flow cell was conducted on a confocal microscope Zeiss LSM 780 with a ×40 objective (Plan-Apochromat, NA 0.95, Korr). This microscope is equipped with a motorised stage (1300×100DC, Carl Zeiss). Thirty-six positions were selected along the chamber for real-time imaging. These defined positions were accurately recorded in the microscope software (Zen 2011) and the intersection point of the two inlets was defined as the zero reference point in the *x-y* plane (Figure 1a and Supplementary Figure 1b). Z-stacks of the biofilm were acquired by using 488 nm excitation wavelength and the GFP emission was detected at 493–598 nm. Z-stacks of 12 slices, 14 slices, 14 slices and 16 slices all at 0.78 μm intervals were used for flow rates 0.1 ml h^−1^, 0.5 ml h^−1^, 1.5 ml h^−1^ and 4.0 ml h^−1^, respectively. The collection of all the images at the 36 positions in an imaging cycle was carried out over a duration of 7 min (flow rate 0.1 ml h^−1^), 8 min 12 s (flow rates 0.5 ml h^−1^ and 1.5 ml h^−1^) and 9 min 20 s (flow rate 4.0 ml h^−1^). The imaging cycles were initiated every 10 min over the duration of the experiments; 8 h 20 min for flow rate 0.1 ml h^−1^, 10 h 20 min for flow rates 0.5 ml h^−1^ and 1.5 ml h^−1^, and 12 h 50 min for flow rate 4.0 ml h^−1^.

### Quantification of three-dimensional biofilm confocal images

For the quantification of biofilm development, we used the three-dimensional stacked images and computed biofilm biovolume by using Imaris (Bitplane, Zurich, Switzerland). We calculated biofilm clustering at 36 positions using the surface segmentation algorithm of Imaris. The biovolume of individual biofilm clusters *i* at position *p* at imaging cycle *n* is defined as *V*_*pni*_. The segmentation parameters used for the above computation were defined as: (a) absolute intensity threshold of 10; and (b) minimum object size of three voxels (each voxel is a cuboid of 0.42 μm×0.42 μm×0.78 μm). The computed biovolume is defined to be the volume of the bacteria cells excluding the additional volume of the extracellular polymeric matrix.

*V*_*pn*_—total biovolume per imaging window at position *p* and imaging cycle *n*, was calculated by summing the biovolume of all the clusters in the imaging window of 212.55 μm×212.55 μm determined by the image acquisition parameters and the specific objectives used.

(1)Vpn=∑i=1NpnVpni

where *p* is the position (*p*=1 to 36), *n* the imaging cycle number (*n*=1 to 47 for flow rate 0.1 ml h^−1^, *n*=1 to 62 for flow rate 0.5 ml h^−1^, *n*=1 to 60 for flow rate 1.5 ml h^−1^ and *n*=1 to 73 for flow rate 4.0 ml h^−1^ (see Supplementary Table 3 for the conversion between actual experiment time *t* and imaging cycle *n*)). *N*_*pn*_ is the total number of clusters in the imaging window at position *p* and imaging cycle *n*. *N*_*pn*_ is varied at different positions and different imaging cycles.

To allow for comparison between different positions at each flow rate, *V*_*pn*_ was normalised against the total biovolume $\left(V_{pn_{a}}\right)$ at the respective position using a reference time point at 2 h (i.e., imaging cycle *n*_*a*_) in all experiments. Time point 2 h was chosen because the bacteria are permanently attached to the surface by that time. The number of clusters at this time was taken as the number of initially attached cluster, *N*_*ap*_. The normalised total biovolume at position *p* and imaging cycle *n* (*V*norm_*pn*_) is calculated as follows:

(2)Vnormpn=VpnVpna

The apparent growth rate at position *p* was assumed to follow an exponential equation described as:

(3)Vpn(t)=Vpn0egpt

where *V*_*pn*_(*t*) is the total biovolume at position *p* at time *t* (i.e., imaging cycle *n*), $V_{pn_{0}}$ is the initial total biovolume at position *p (*i.e., at imaging cycle *n*_*0*_), *g*_*p*_ is the apparent growth rate at position *p* and has a positive value, *t* is the time. The average growth rate at position *p* $\left(\overset{¯}{g_{p}}\right)$ was calculated by fitting *V*_*pn*_ during the period from the start of experiment (*n*_*0*_=1) to the time of maximal observed growth at that position; at imaging cycle *n*_*p*max_ is the cycle at which total biovolume reached its maximal value.

The doubling time at position *p*, $t_{d_{p}}$, was calculated by:

(4)tdp=ln2gp¯

The distribution of cluster sizes at position *p*, at imaging cycle *n*, was plotted by sorting *V*_*pni*_ in ascending order against the total number of clusters at that position *N*_*pn*_ (with *p* being the position, *p*=1 to 36).

The bubble plot represents the cluster size and its spatial distribution at each defined time point. Each bubble represents an individual cluster *i* at position *p* taken in imaging cycle *n*. Its diameter, *D*_*pni*_, was computed from *V*_*pni*_ by assuming the cluster as a sphere as follows:

(5)Dpni=6Vpniπ3

The centre of the bubble was the centroid of the respective clusters.

A summary of the experimental parameters and specifications of image acquisition is presented in Supplementary Table 1.

## Figures and Tables

**Table 1:** Comparison of dispersal time at position 12 of various experiments

Q* (ml h^−1^*)	*Run 1*		*Run 2*			
	$\overset{¯}{t12disp1}\pm \sigma 1$ *(min)*	$\sigma 1/\overset{¯}{t12disp1}$ *(%)*	$\overset{¯}{t12disp2}\pm \sigma 2$ *(min)*	$\sigma 2/\overset{¯}{t12disp2}$ *(%)*	*Δ*_*12*_ *(min)*	$\Delta 12/\overset{¯}{t12disp1}$ *(%)*
0.1	370±6	1.6	387±0	0.0	17	4.6
0.5	432±6	1.2	438±0	0.0	6	1.4
1.5	472±6	1.3	468±0	0.0	4	0.8
4.0	539±10	1.9	—	—	—	—

$\overset{¯}{t12disp}$ is the average of dispersal time at positions 12 a–c. Δ_12_ is the difference of dispersal time at positions 12 between the two runs of the same flow rate.
