# Supplementary material for: Biofilm development of an opportunistic model bacterium analysed at high spatiotemporal resolution in the framework of a precise flow cell
Source: NPJ Biofilms Microbiomes. 2016 Oct 19;2:16023–. doi: 10.1038/npjbiofilms.2016.23 (PMC5515269; doi:10.1038/npjbiofilms.2016.23)
Supplement: Supplementary Information [file npjbiofilms201623-s1.doc]

**SUPPLEMENTARY INFORMATION**

**Supplementary Note 1. Assembly of flow cell system**

Glass coverslips on which biofilms developed, other components of the flow cell, valves and PTFE tubes were first soaked in ethanol 70% v/v (Merck) in DDW for 15 min, followed by drying by using an air gun. The cleaned coverslips and acrylic plates carrying the channel, all were disposable in each experiment, were additionally UV-treated (CL-1000 UV cross linker, 254 nm, UVP Inc., CA, USA) for 30 min to disinfect potential biological contaminants. The flow cell was first assembled inside a biosafety cabinet. The list of components and assembly of the flow cell were well documented in our patent[^1^](#_ENREF_1). In contrast to most existing flow cells, our flow cell was specifically designed to have a removable coverslip fixed to the flow cell without the need of permanent bonding (for example adhesive or thermal bonding). Subsequently, the two three-way valves, the effluent containers and the syringe pump were connected to the assembled flow cell. The system was then mounted onto a microscope, either on an inverted microscope for flow velocity measurement or on a confocal microscope for live imaging of biofilm development (Supplementary Fig. 1a).

**Supplementary Note 2. Particle image velocimetry (PIV) measurement**

PIV is a technique for flow visualization and flow velocity measurement. In PIV, a fluid is seeded with tracer particles and its flow velocity is calculated from the particles’ motion. Briefly, consecutive images (i.e. an image pair) separated by a time lag of the particles-seeded fluid flow are taken. Cross-correlation of the two images is then conducted to obtain the flow velocity of the fluid.

We seeded a water-glycerol solution, particularly 20% w/w glycerol (Sigma-Aldrich) in water, with fluorescent polystyrene microspheres (3.2 µm diameter Fluoro-Max Dyed Red Aqueous Fluorescent Particles, Thermo Scientific Inc.) at microsphere concentration of 0.1% w/w. The fluid was infused into the flow cell at low flow rate Q = 0.1 ml h^-1^ per inlet and at high flow rate Q = 1.5 ml h^-1^ per inlet with the aid of a syringe pump. The motion of the microspheres in the channel was captured using a high speed camera (Photron FASTCAM SA-5, Japan) on an inverted epi-fluorescence microscope (Nikon Ti-eclipse with Nikon Intensilight light source and Nikon TRITC filter cube, Japan) at magnification of 10x (Nikon Plan-Fluor 10x objective lens with N.A. 0.30, Japan). We positioned the focal plane of the objective at the half-depth of the channel to measure the mid-plane velocity. The field of view of the high speed camera was 2,048 µm x 752 µm and the flow velocity along the central region of the flow cell (from *x* = 0 mm to *x* = -12 mm) was sequentially measured over 9 imaging positions. Each imaging position was measured for 120 s and then was offset by 1,250 µm along the *x* direction, resulting in an overlapping region of 798 µm wide between two consecutive positions.

The selection of optimum PIV image acquisition parameters, namely, time between image pair, image pair acquisition frequency and exposure time, is dependent on the flow velocity. Therefore, the image acquisition parameters used for low and high flow rates were different (Supplementary Table 1). The flow velocities were computed from the acquired images using a PIV program written in Matlab (Mathworks, MA, USA) that was adapted from OpenPIV[^2^](#_ENREF_2). The time-averaged velocities at each position were calculated and the flow velocity along the centerline of the flow cell was interpolated from the 9 positions. The measured velocities were then compared with the simulated velocity (Figs. 1e – f).

**Supplementary Note 3. Calculation of theoretical cluster size distribution**

Theoretical cluster size distribution was computed by assuming each microcluster that was attached at *t* = 2 h (at cycle *n_a_*) developed clonally following exponential growth equation as follows:

| $V_{pni\text{ }}(t)=V_{pn_{\text{ 0}}i}\text{ }e^{\overline{g_{p}}\text{ }t}$ | (1) |
| --- | --- |

Each microcluster’s biovolume was assumed to increase exponentially based on the average growth rate $\overline{g_{p}}$ at their respective position *p*. The biovolumes were computed up to the time of maximal observed growth corresponding to the imaging cycle *n_pmax_*.

The calculated theoretical cluster size distribution (grey) at position 7a was plotted together with the experimental cluster size distribution (black) at the same position for four flow rates up to the time of maximal observed growth in Supplementary Video 8b.

| **(a)**  **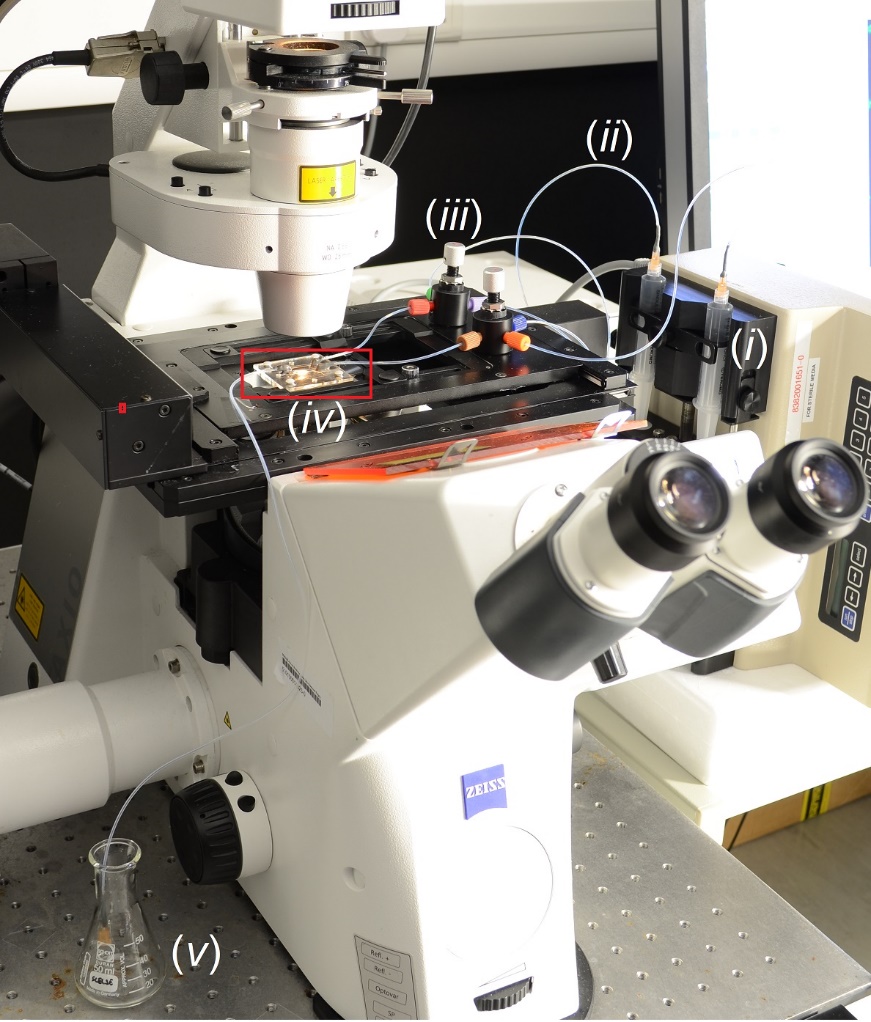** |
| --- |
| **(b)**  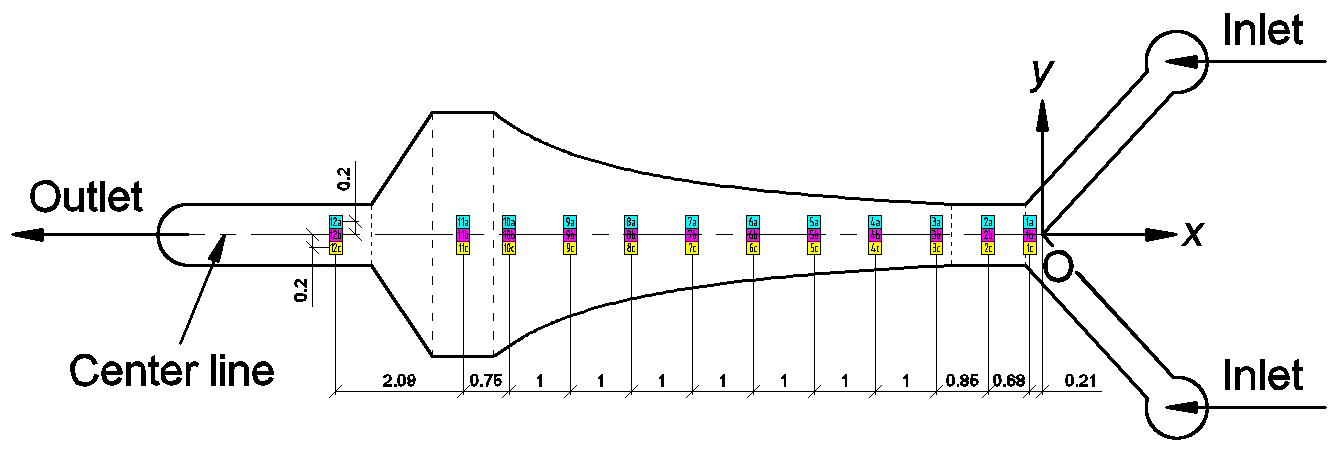 |
| Supplementary Figure 1. (a) Set-up of flow cell system on a confocal microscope Zeiss LSM 780; (i) Syringe pump (ii) Tubing (iii) Valves (iv) Flow cell (v) Effluent collector; (b) Profile of hyperbolic channel with center-to-center dimensions between 36 imaging positions. All units in mm. |

| **(a)** | **(b)** | | **(c)** |
| --- | --- | --- | --- |
| 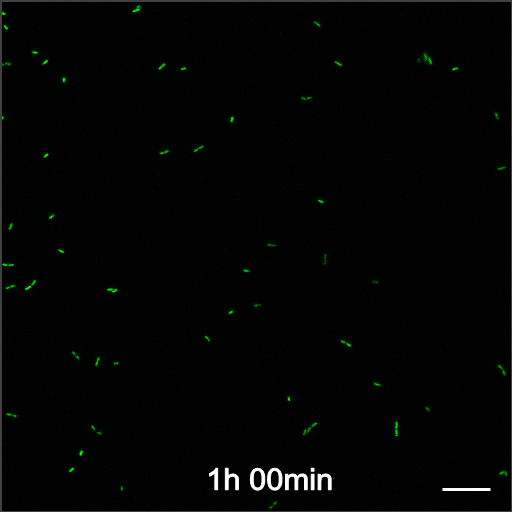 | 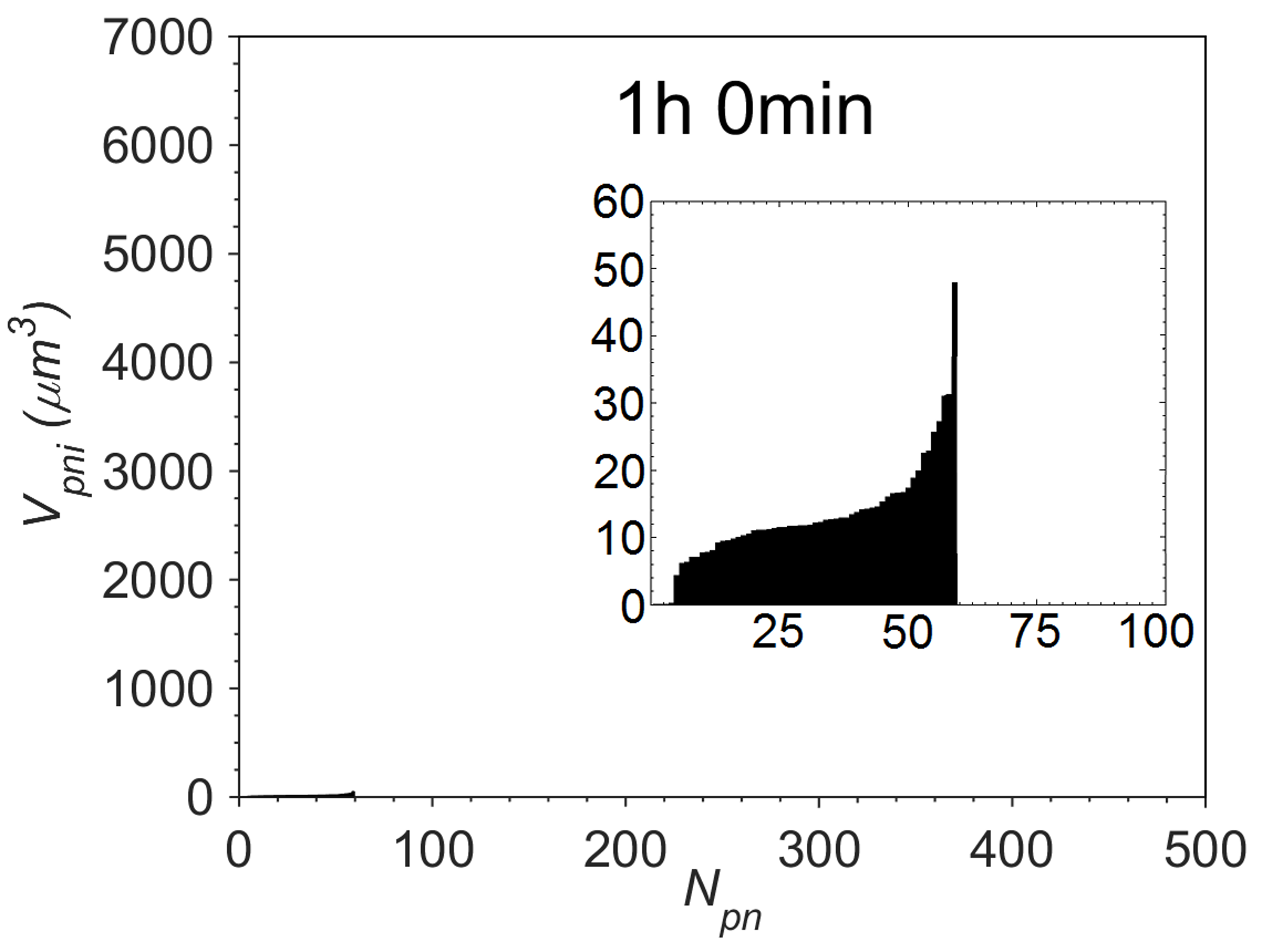 | | 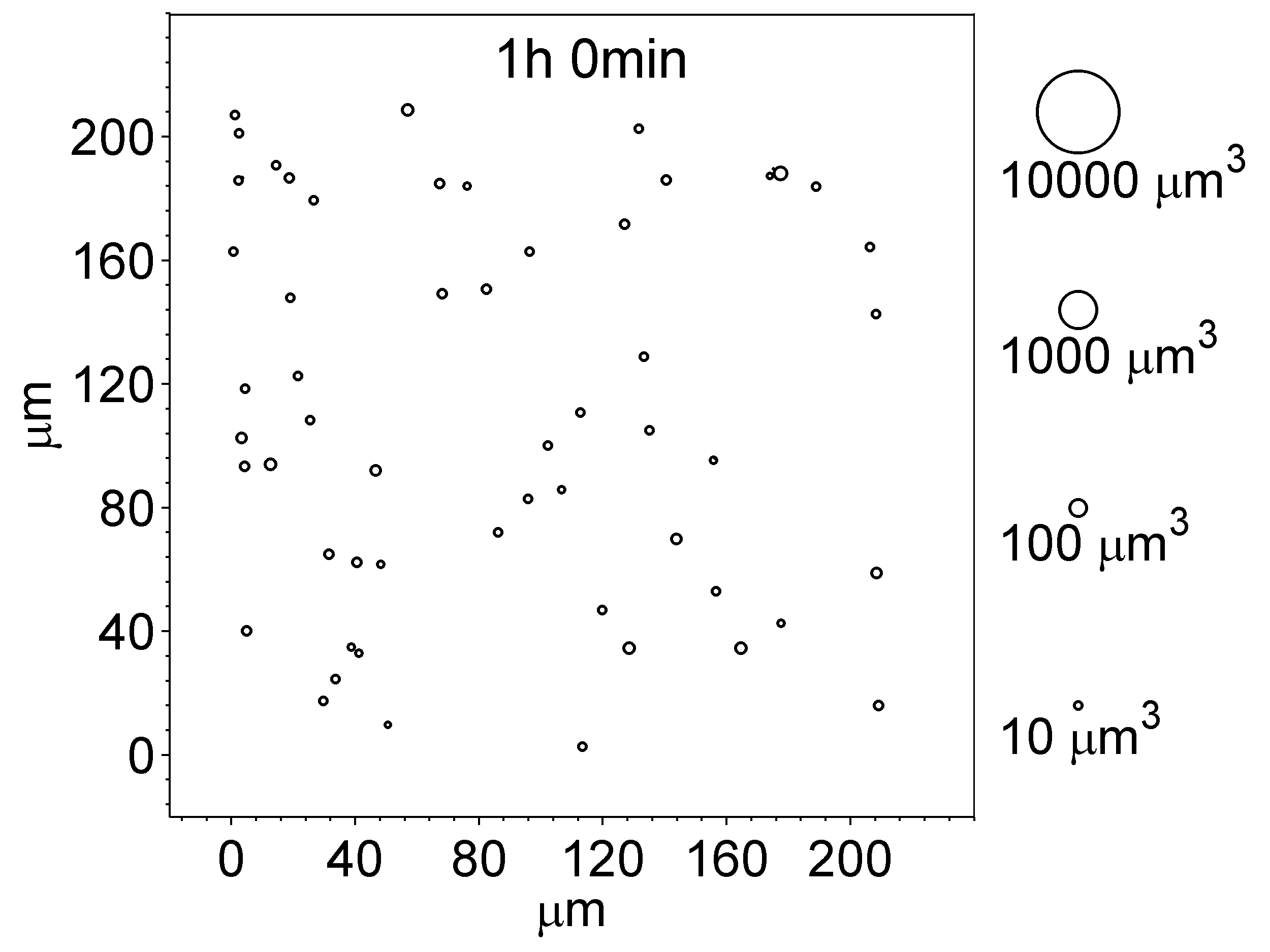 |
| 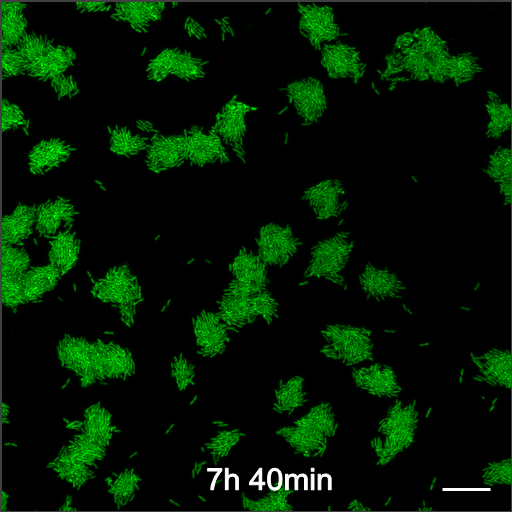 | 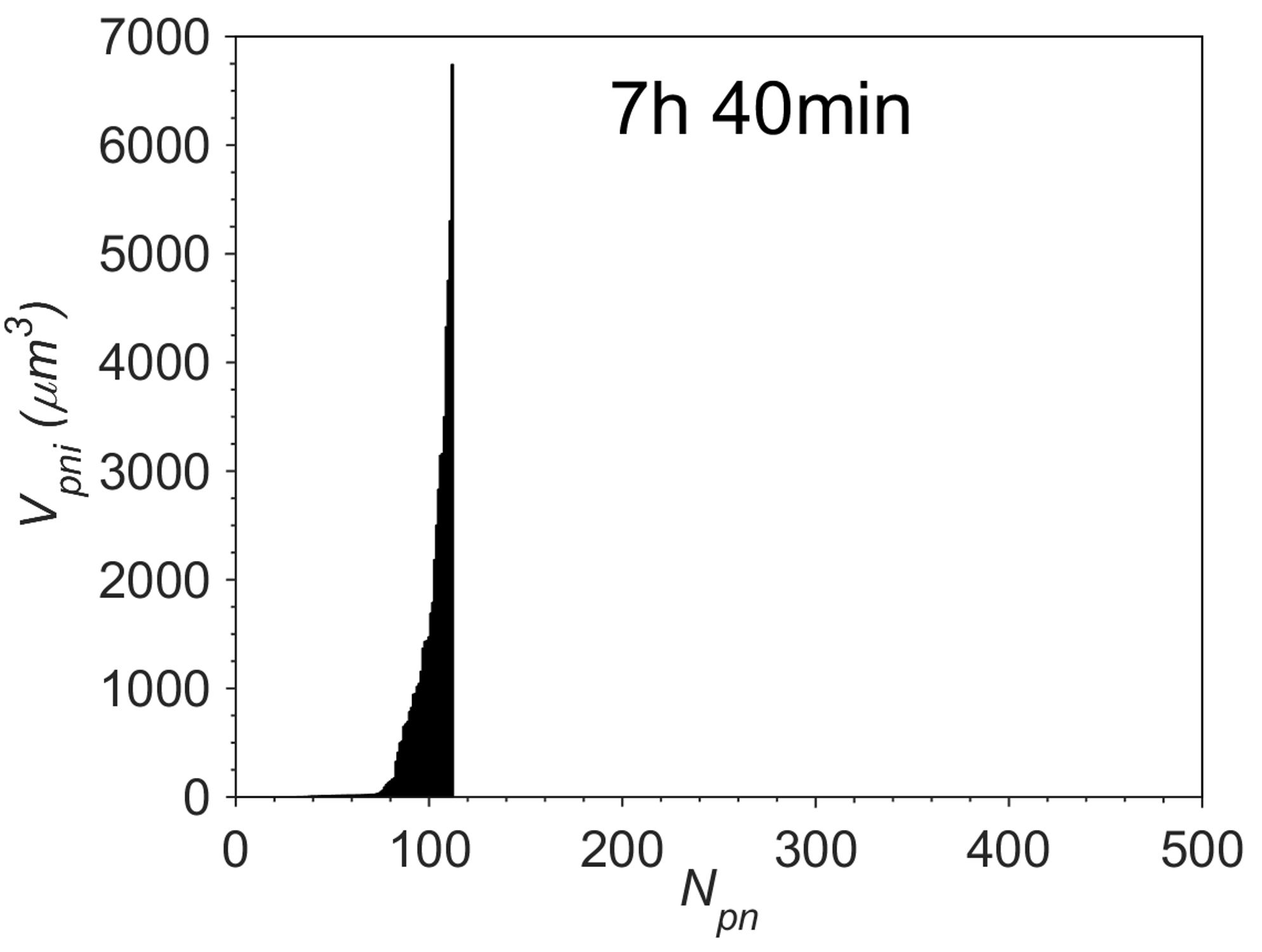 | | 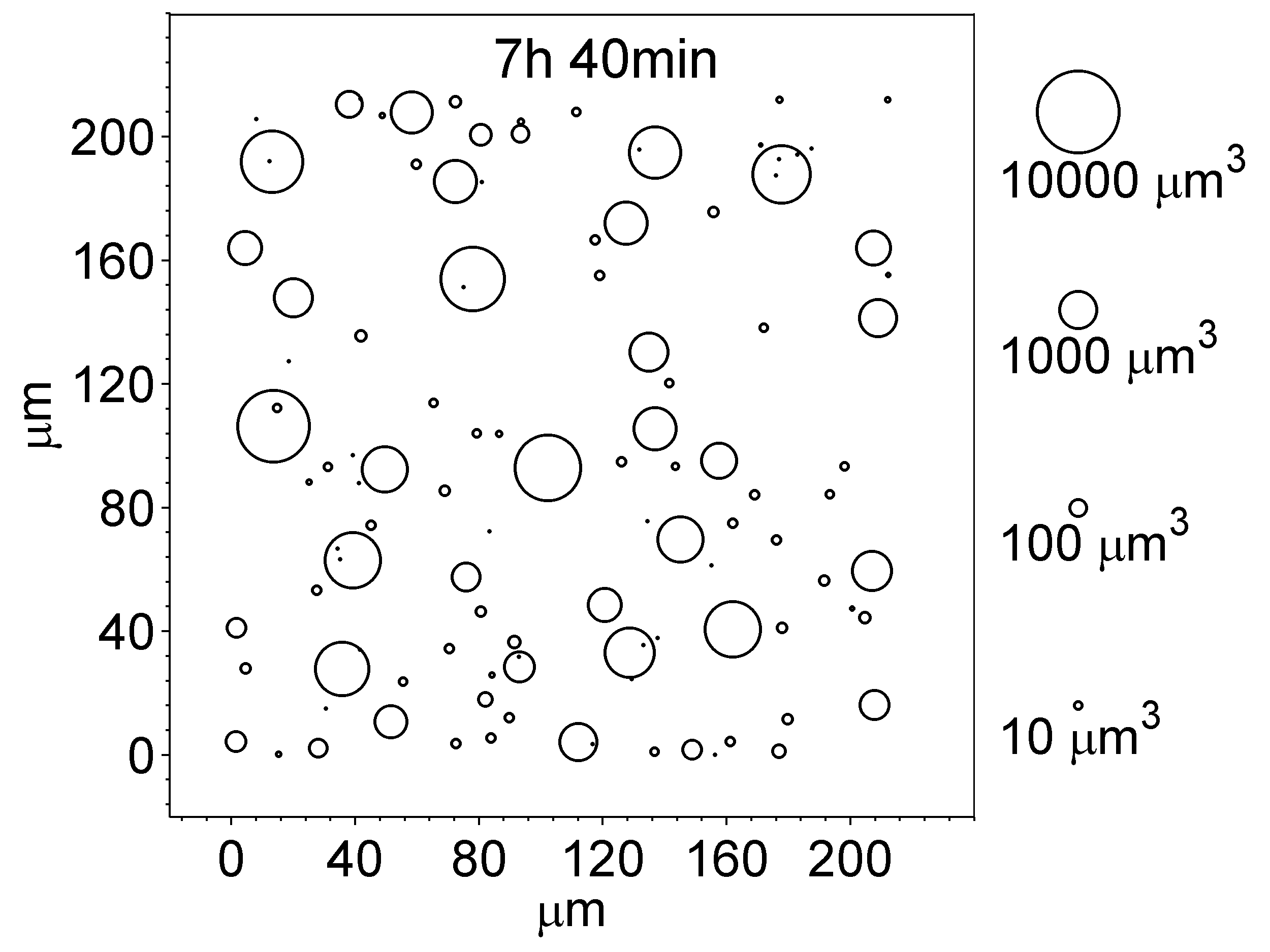 |
| 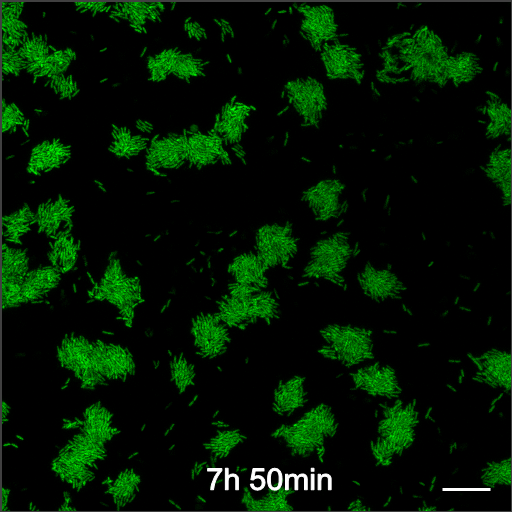 | 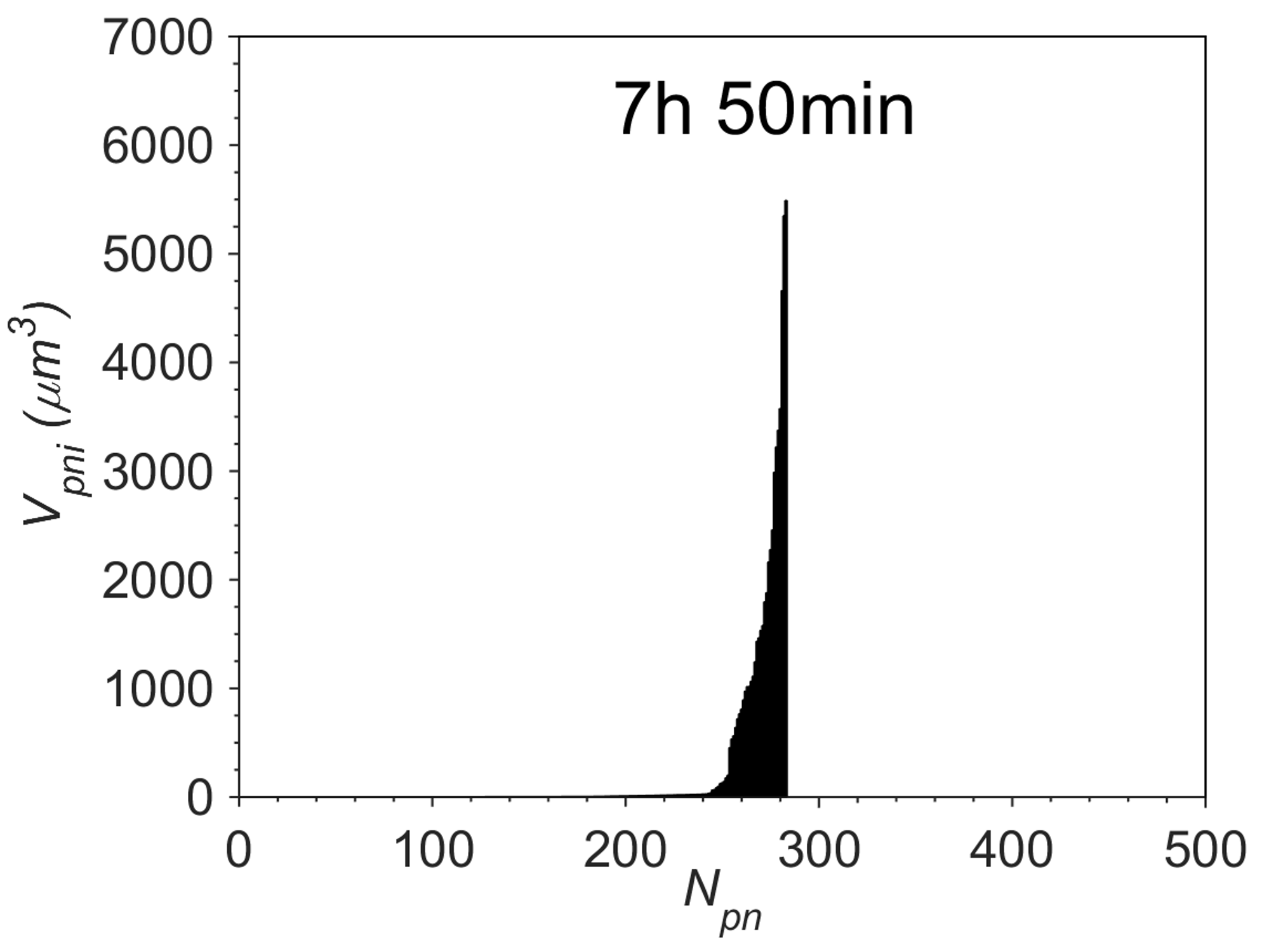 | | 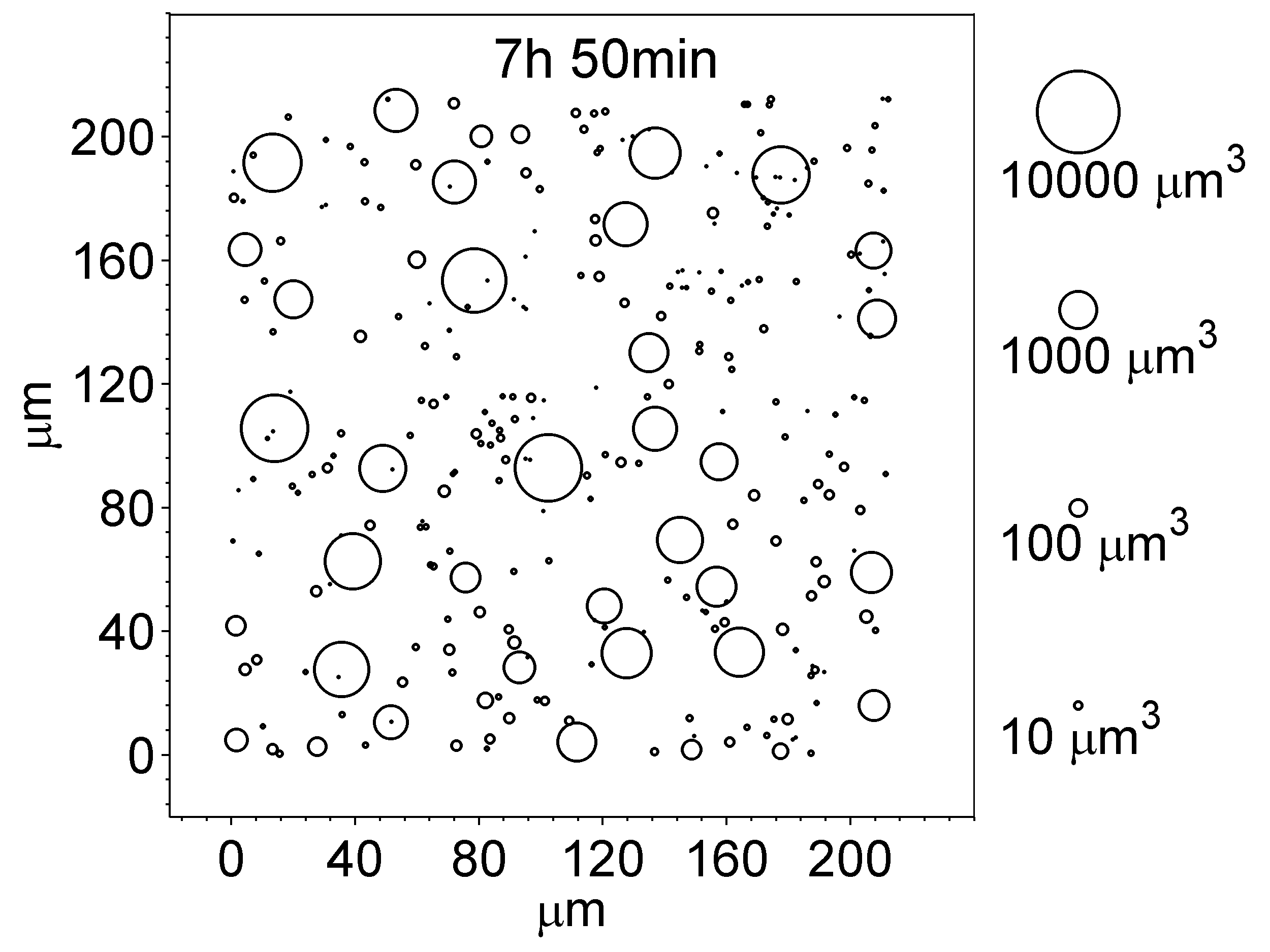 |
| 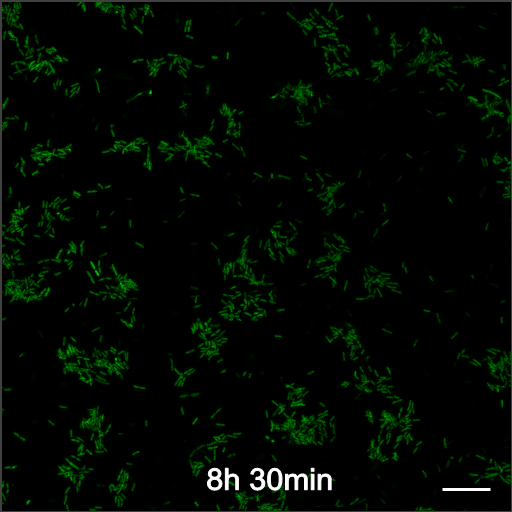 | 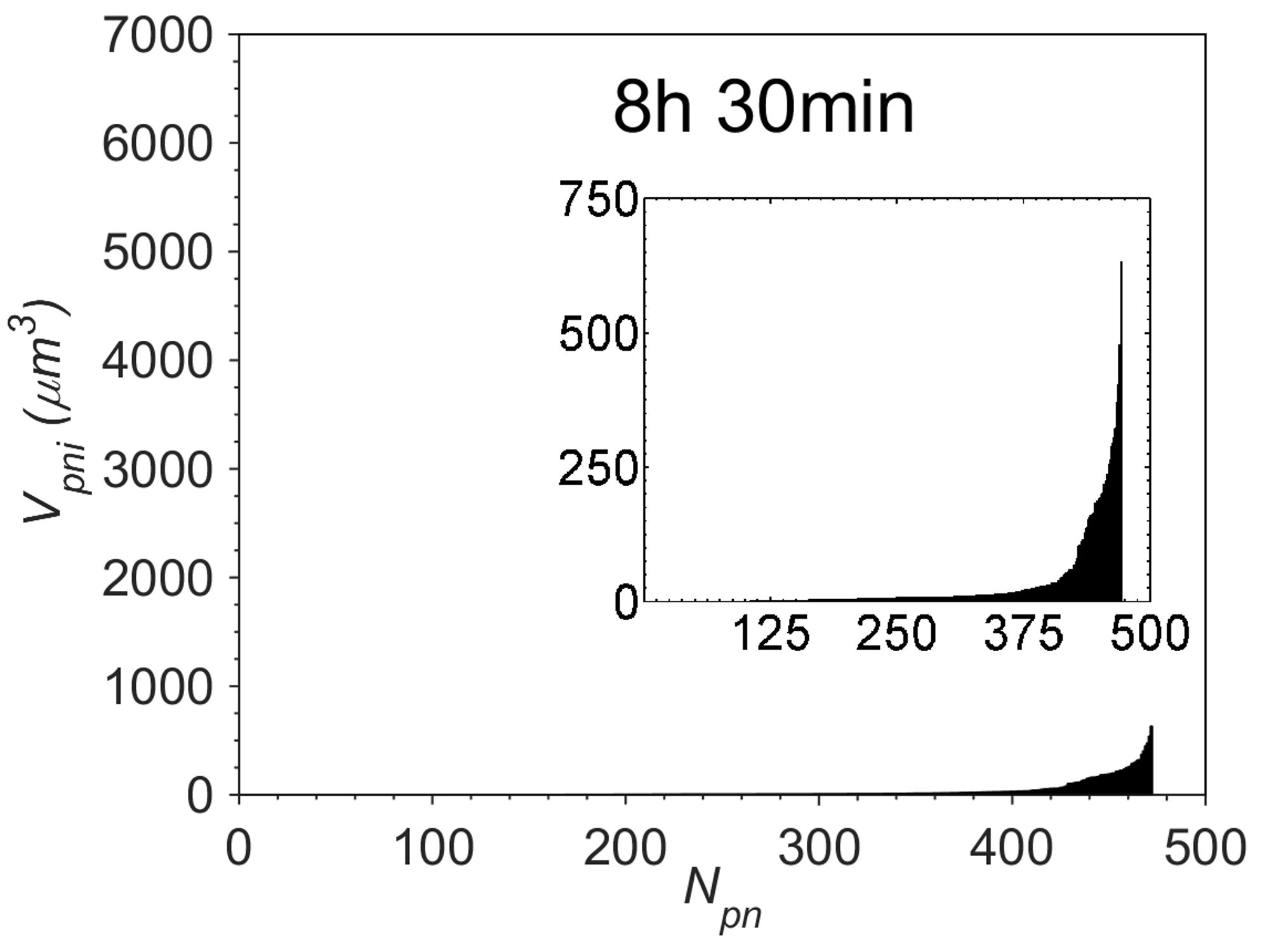 | | 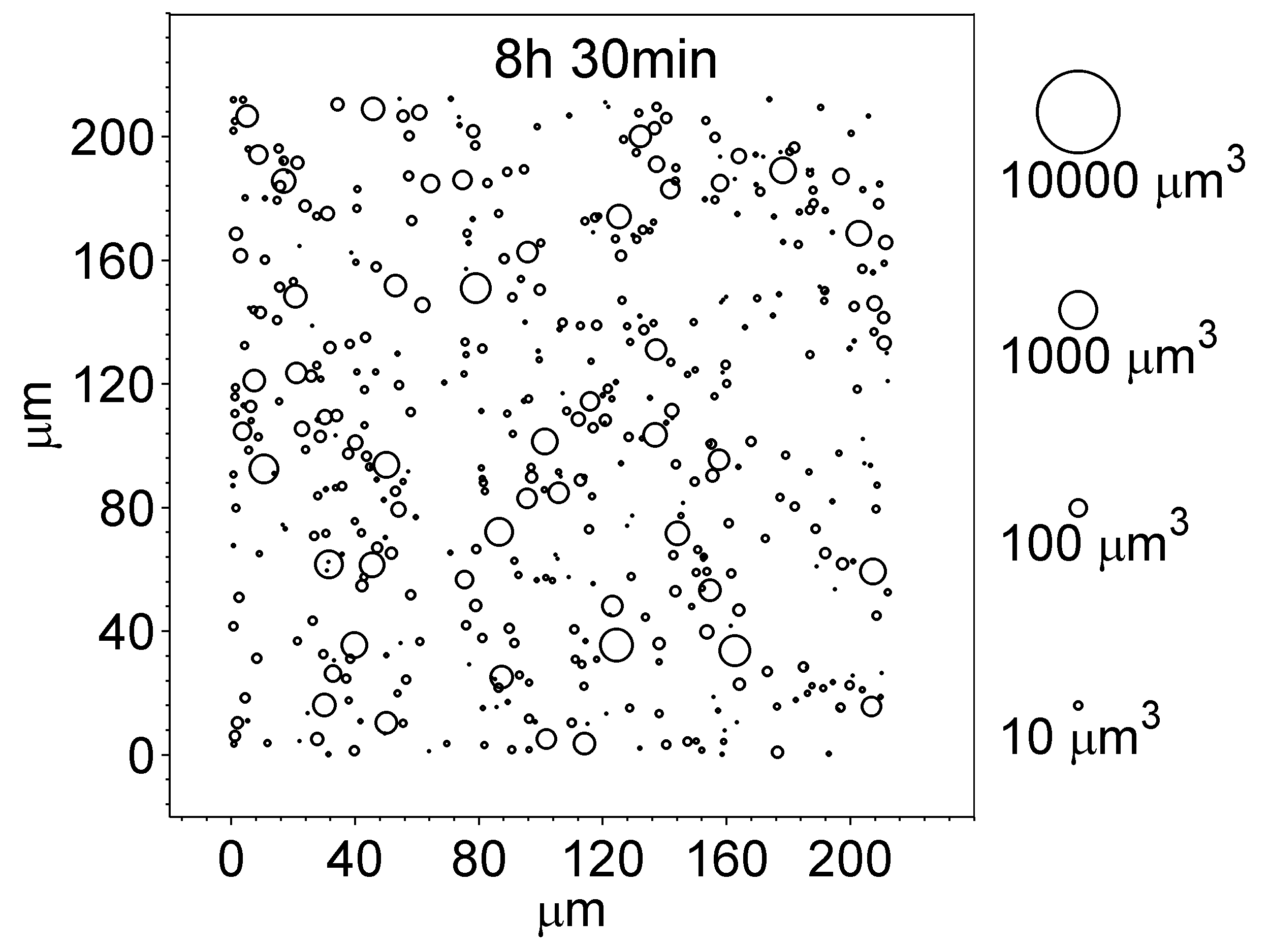 |
| 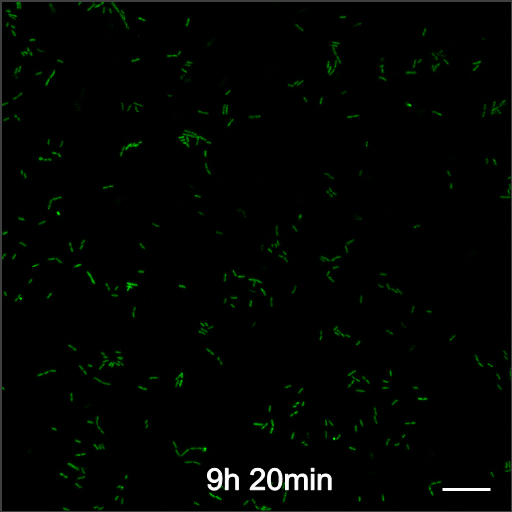 | 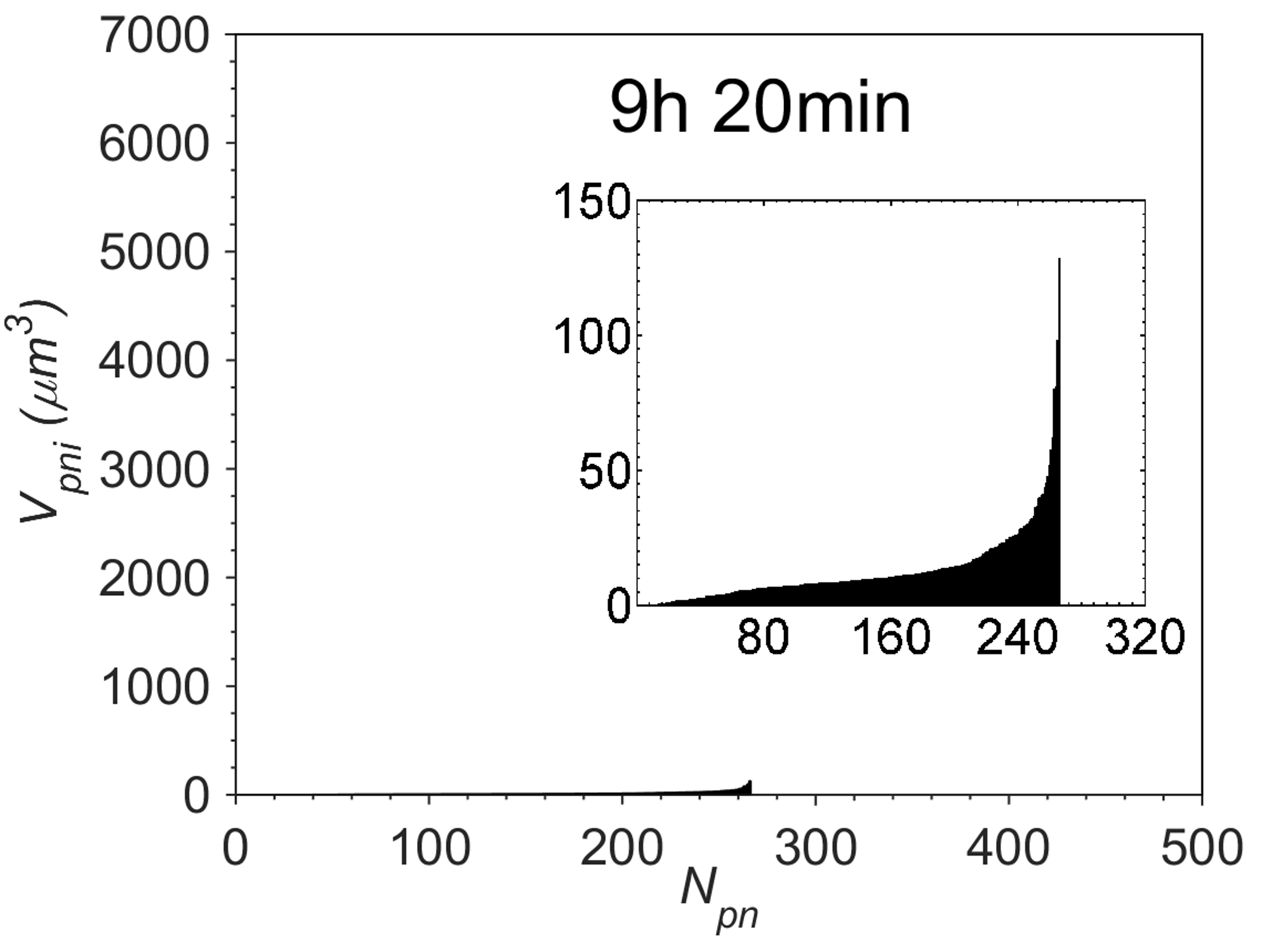 | | 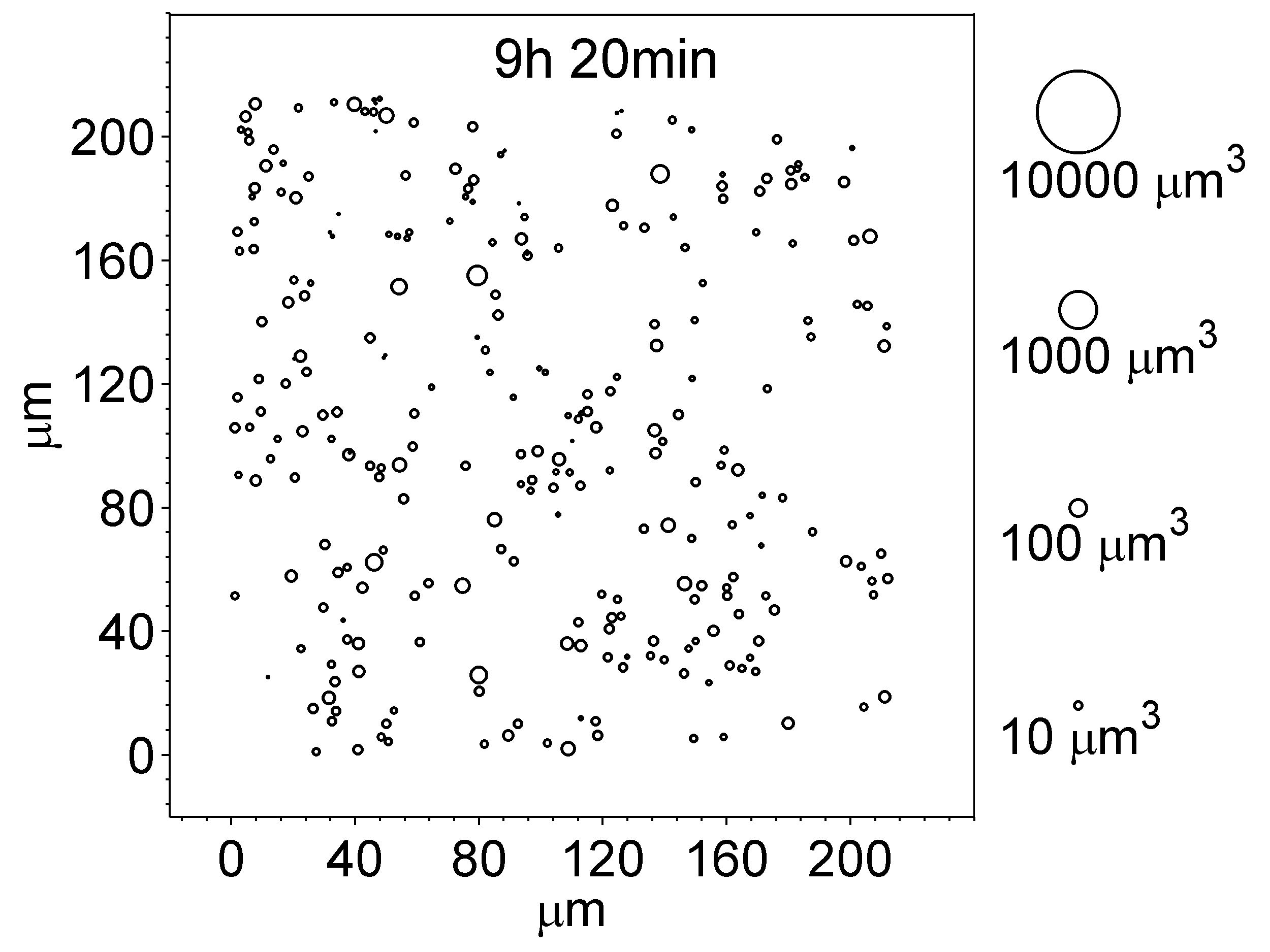 |
| **(d)**  **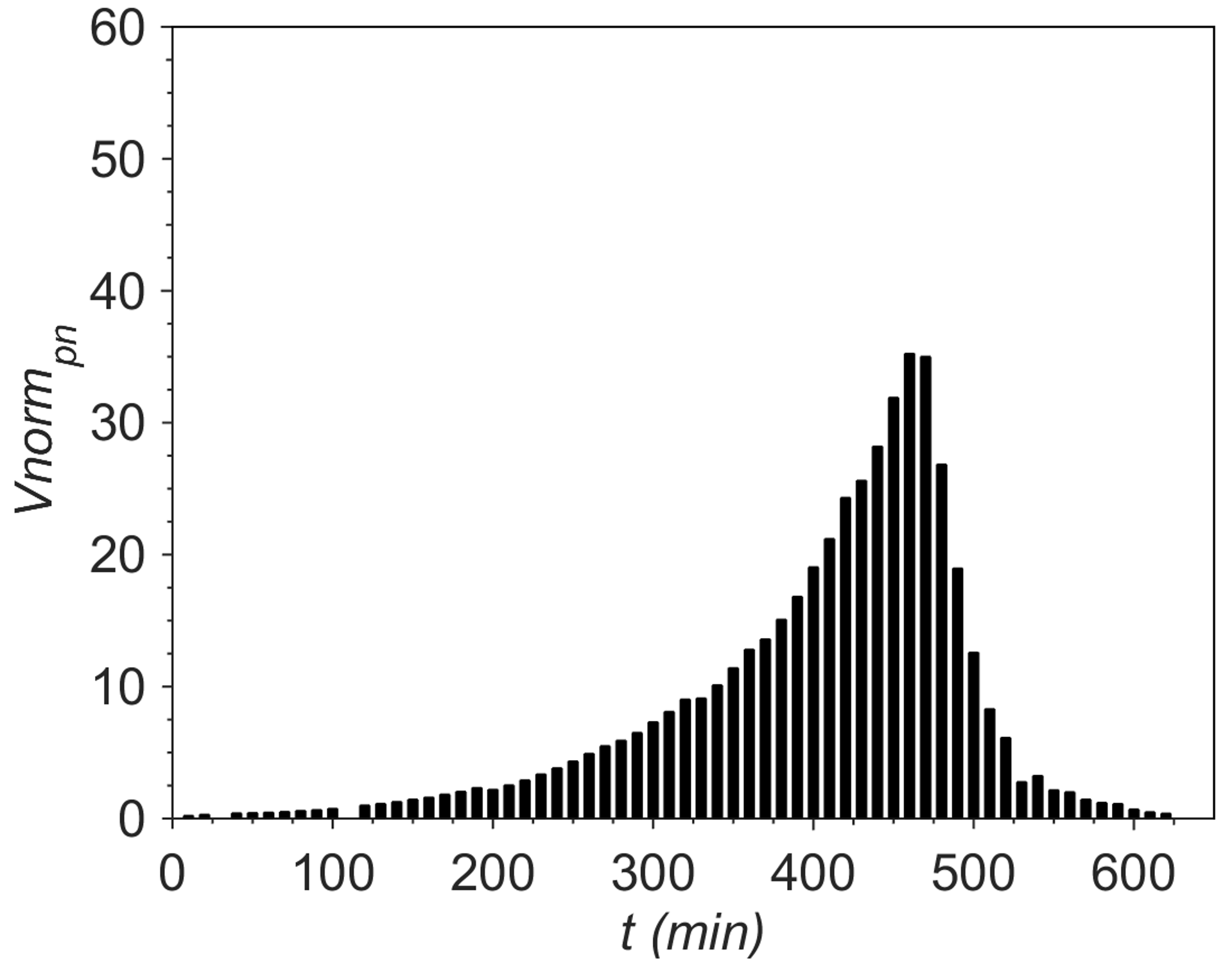** | |  | |

Supplementary Figure 2 – Dynamics of *P. putida* OUS82::GFP cluster formation and dispersal at position 7a at significant time points under high flow rate Q = 1.5 ml h^-1^ per inlet. (a) Confocal images of the initial biofilm development: 1 h (initial attached bacteria), 7 h 40 min (maximal growth of biofilm cluster), 7 h 50 min (commencement of dispersal), 8 h 30 min (dispersal of biofilm) and 9 h 20 min (fully dispersed biofilm, certain *P. putida* that remained on the surface resembled filaments). Scale bar: 20 µm. Supplementary Video 7 shows the 10 h 20 min-period time lapse of the biofilm development at this position. A three-dimensional view and a cross-section view of the confocal image at 7 h 40 min are shown in Supplementary Figs. 3c – d. (b) Cluster size distribution at position 7a corresponding to the confocal images in (a). The *y*-axis is the individual cluster biovolume, *V_pni_*, while the *x*-axis is the total number of cluster present in the imaging window, *N_pn_*. The entire distribution of cluster size over 10 h 20 min at this position is provided in Supplementary Video 8a. (c) Bubble plot of the spatial distribution of *V_pni_* for the corresponding time point in (a). Supplementary Video 9 shows the bubble plot corresponding to the biofilm development at this position over 10 h 20 min. (d) Normalized total biovolume, *Vnorm_pn_*, at position 7a over time. Supplementary Video 10 shows biovolume per imaging window, *V_pn_*, vs *t* for 36 positions at flow rate 1.5 ml h^-1^ per inlet.

| **(a)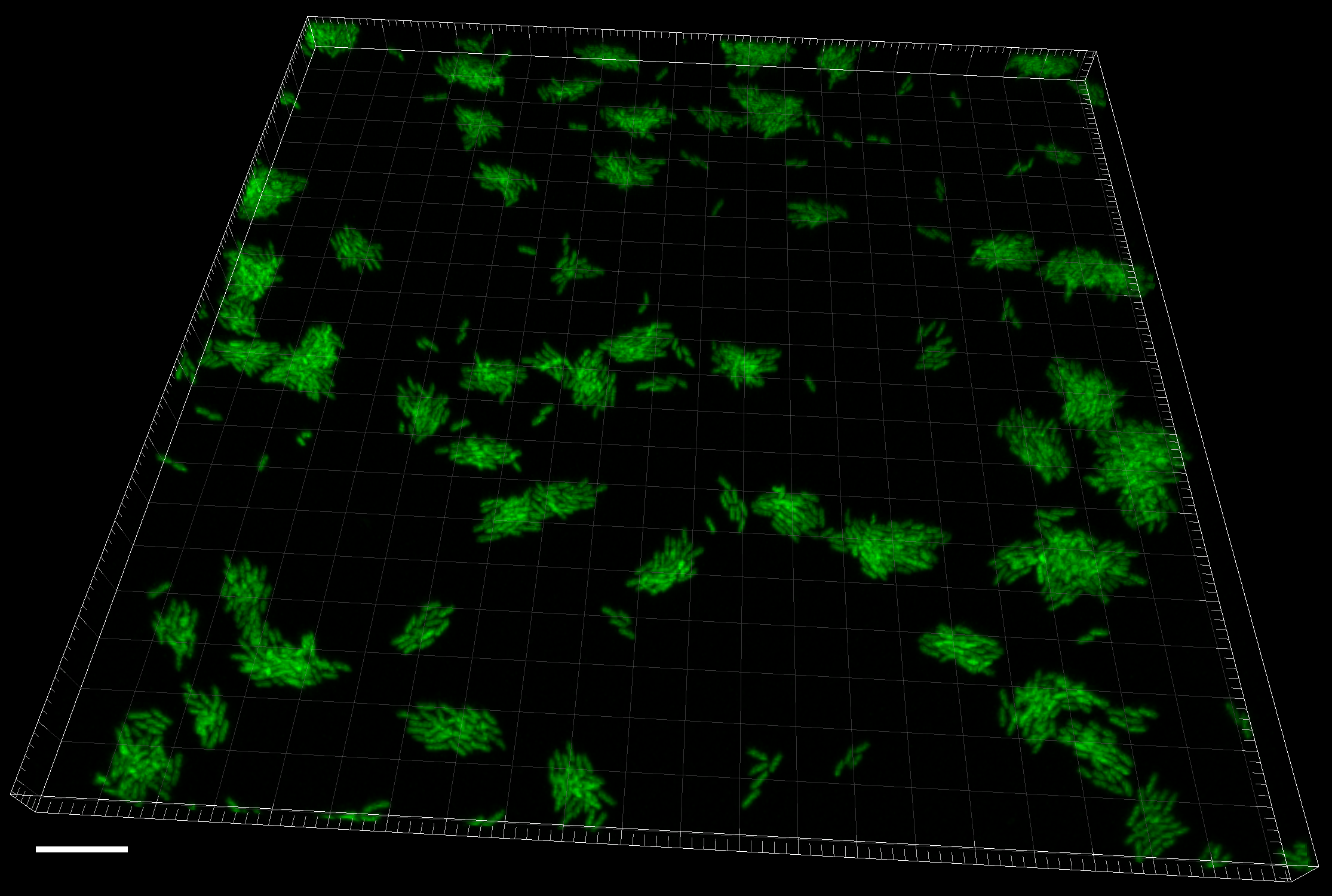** | **(c)**  **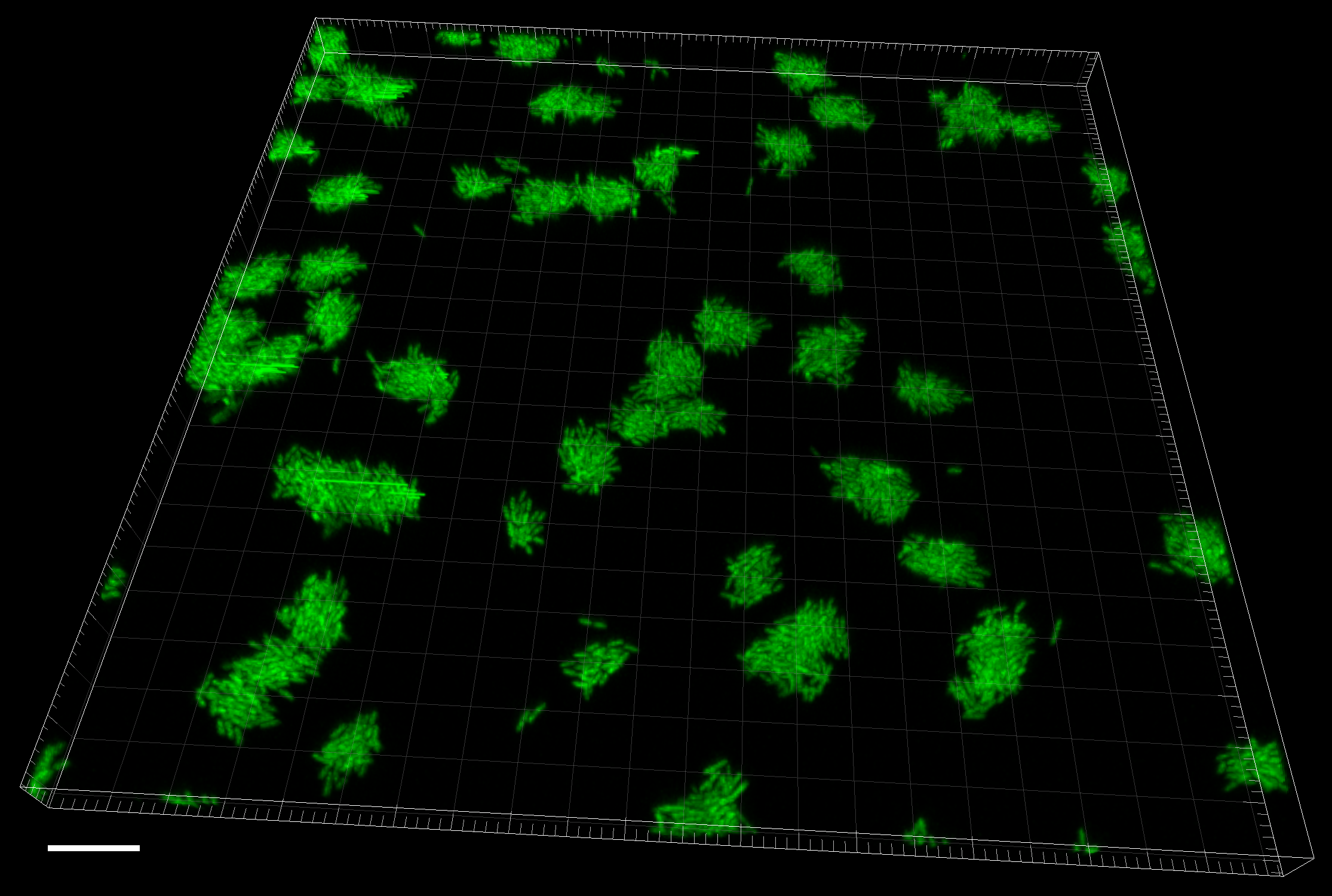** |
| --- | --- |
| **(b)** | **(d)** |
| 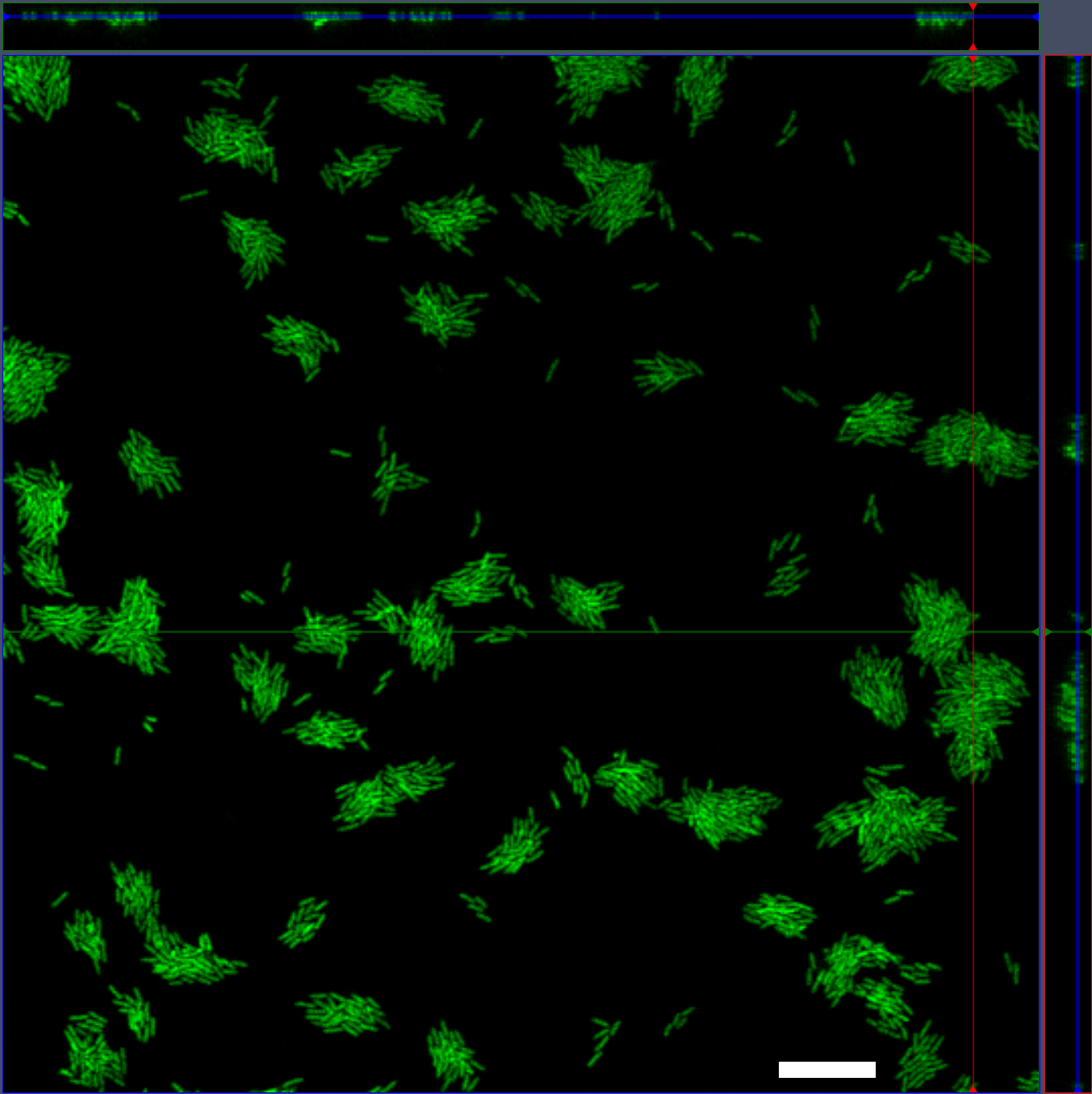 | 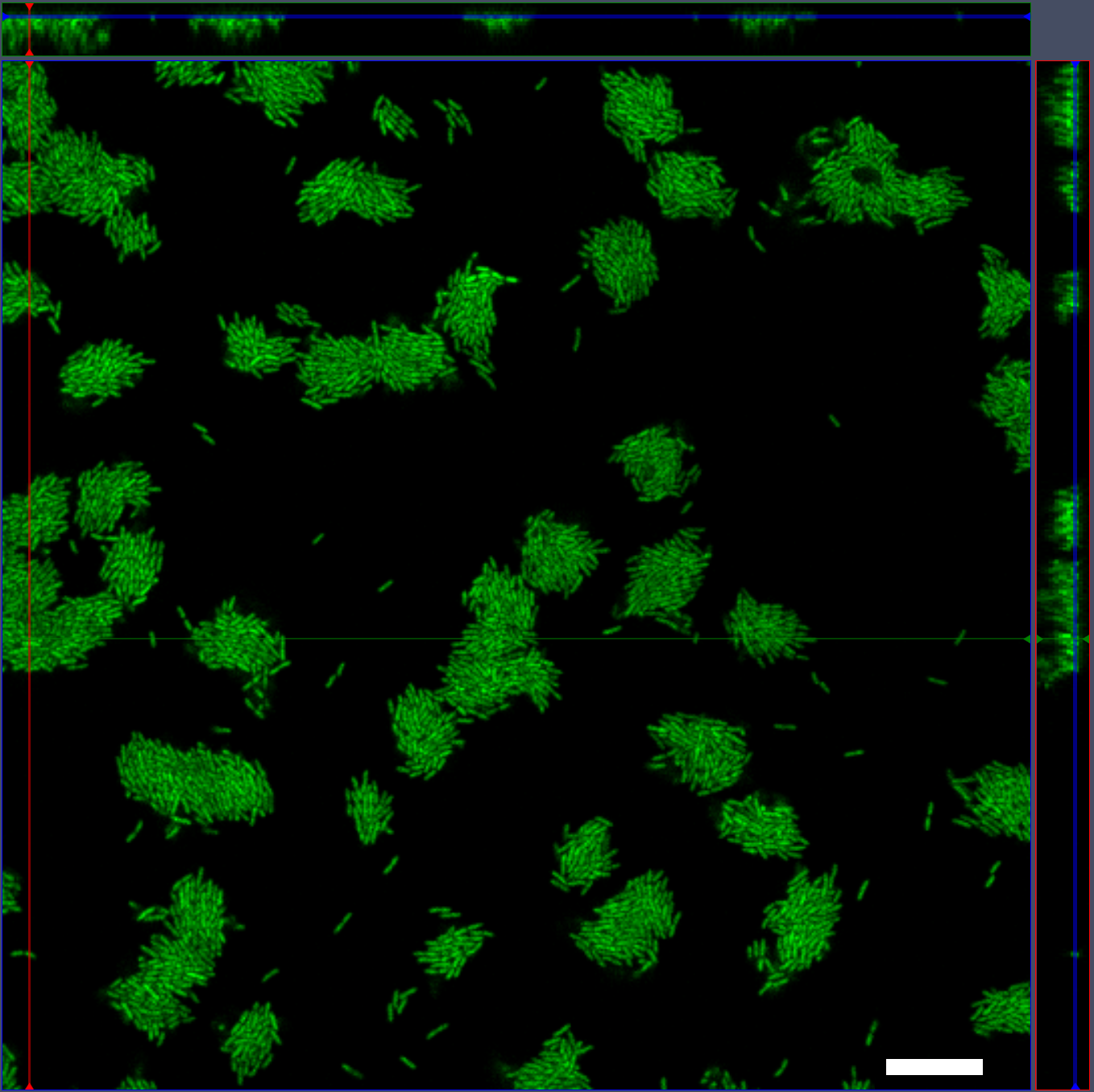 |
| Supplementary Figure 3. Microcolonies structure of *P. putida* biofilm developed at position 7a under various flow conditions. (a) Three-dimensional confocal image, constructed from a Z-stack of 12 slices at 0.78 µm interval, of biofilm at 6 h 20 min under low flow rate Q = 0.1 ml h^-1^ per inlet. (b) Cross-section view of microcolonies that reached their maximal growth after 6 h 20 min under low flow rate Q = 0.1 ml h^-1^ per inlet. (c) Three-dimensional confocal image, constructed from a Z-stack of 14 slices at 0.78 µm interval, of biofilm at 7 h 40 min under high flow rate Q = 1.5 ml h^-1^ per inlet. (d) Cross-section view of microcolonies at their maximal growth after 7 h 40 min under high flow rate. Magnification: 40x. Scale bar: 20 µm. In the cross-section views, the top and side images represent *x-z* and *y-z* planes respectively. | |

| **(a)**  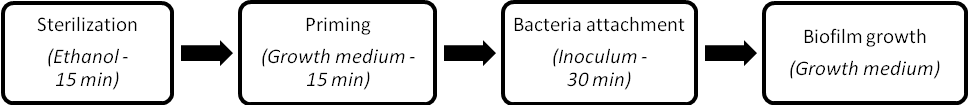 |
| --- |
| **(b)**  **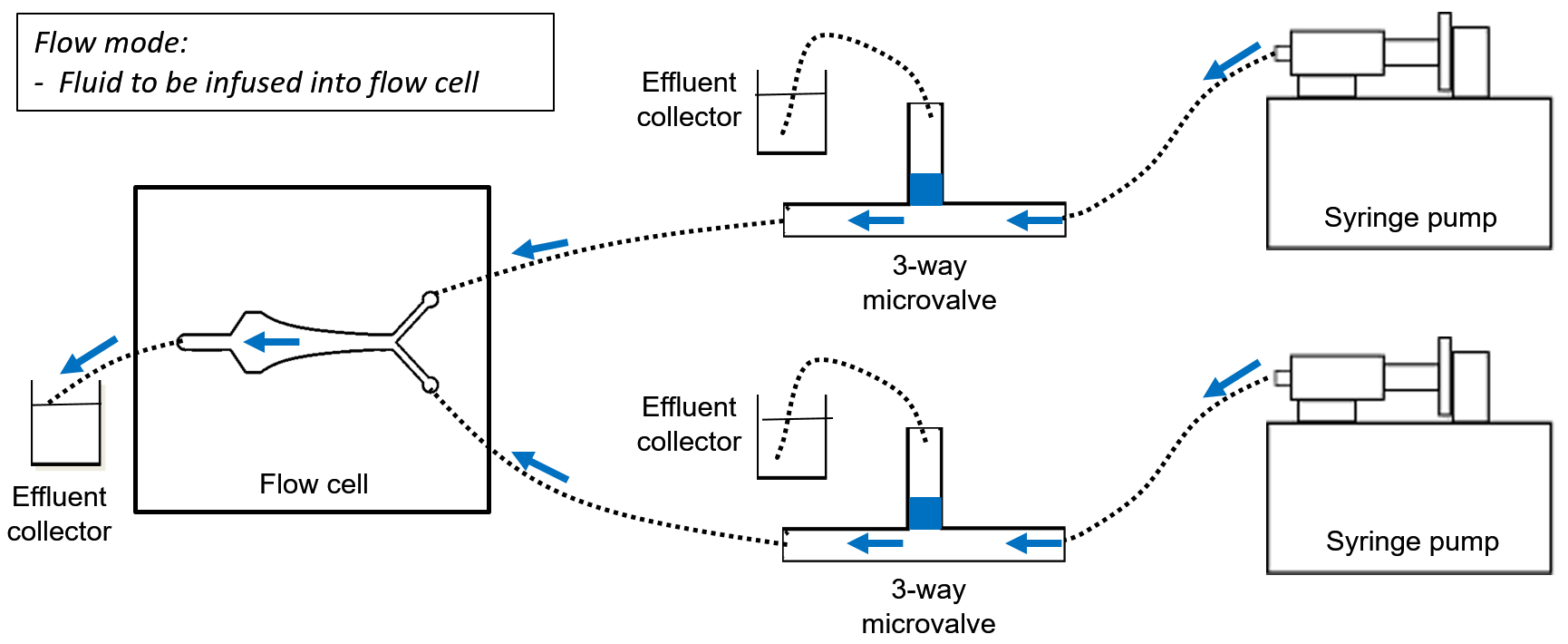** |
| **(c)**  **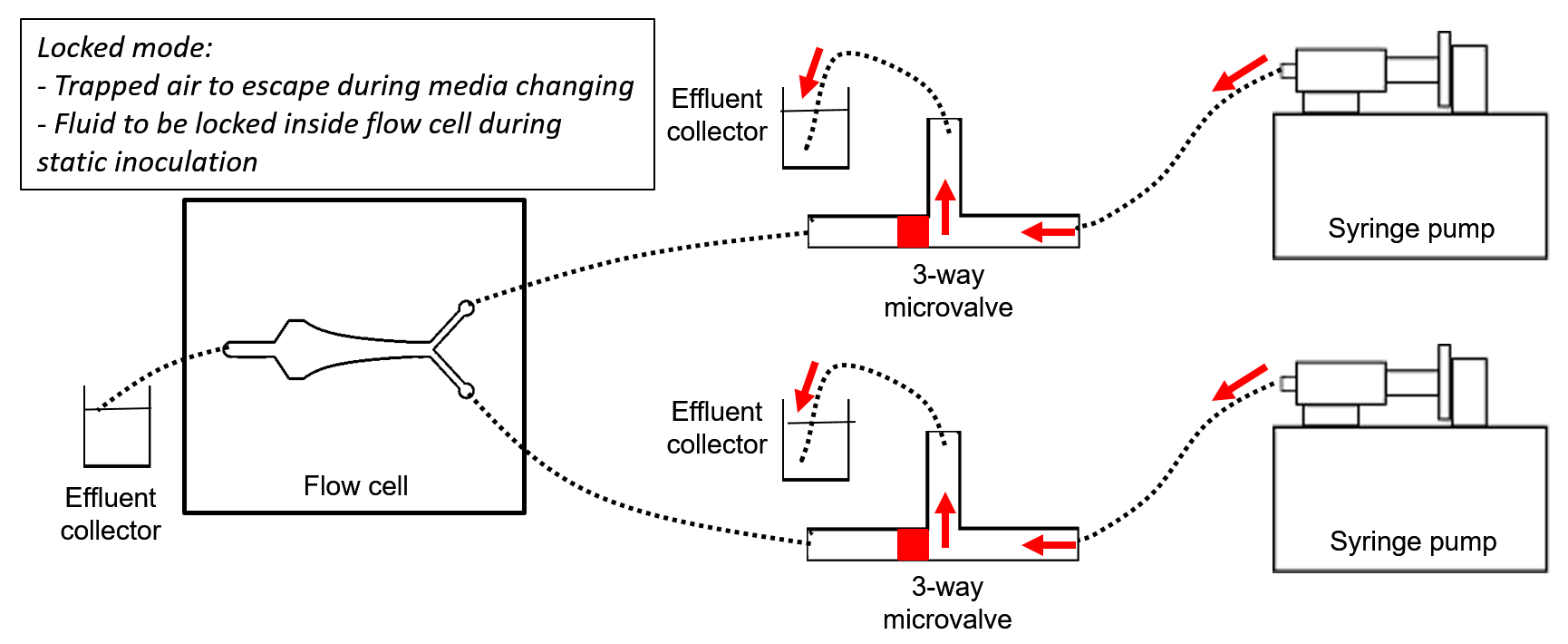** |
| Supplementary Figure 4. Procedure and operation of biofilm growth experiment  (a) Procedure of biofilm growth experiment to minimize risk of contamination; (b) and (c) Set-up of flow cell system with the aid of syringe pumps for precise control of fluid infused and three-way microvalves for flexible switching of various types of media without air trapped inside the flow cell. (b) Flow mode: Fluid from syringes mounted on syringe pumps is infused directly into the flow cell through the two inlets. This position is used during most of the time in an experiment except when the locked mode is in use; (c) Locked mode: Fluid with any trapped air inside due to changing of syringes is stopped before entering the flow cell. Instead, the fluid is directed to the effluent collectors where the portion of fluid with trapped air can be fully disposed. This position can be used during (1) Switching of media infused into the flow cell without air trapped. Media can be ethanol for priming, inoculum, growth media, etc. (2) Static inoculation – Inoculum is first infused into the flow cell by flow mode, then the system is switched to locked mode so that inoculum is locked inside the flow cell for cell attachment onto the coverslip surface. |

Supplementary Table 1 – Summary of experimental parameters and specifications

| **Experiment**   | **Q = 0.1 ml h^-1^** | **Q = 1.5 ml h^-1^** | **Q = 0.5 ml h^-1^** | **Q = 4.0 ml h^-1^** |  | | --- | --- | --- | --- | --- | | Related figures | Figs. 1a – e, 2, 4, 5, 6  Supplementary Figs. 1, 3a – b, 4 | Figs. 1a, b, d, f, 3, 5, 6  Supplementary Figs. 1 – 2, 3c – d, 4 | Figs. 1a, b, d, 5, 6  Supplementary Figs. 1, 4 | Figs. 1a, b, d, 5, 6  Supplementary Figs. 1, 4 | | Related supplementary videos | 1, 5 – 10 | 3, 5 – 10 | 2, 5 – 10 | 4 – 11 | | **PIV measurement** | | | | | | - Microscope | Nikon Ti-eclipse epi-fluorescence inverted microscope | | N.A. | | | - Objective | Plan-Fluor 10x N.A. 0.30 | | | - Camera | Photron FASTCAM SA-5 | | | - Scaling (per Pixel) | 2 µm x 2 µm | | | - Image Size (Pixels) | 1,024 x 376 | | | - Image Size (scaled) | 2,048 µm x 752 µm | | | - Time between image pair | 400 ms | 16 ms | | - Exposure time | 25 ms | | | - Image pair acquisition frequency | - 1. Hz | 5 Hz | | - Number of positions | 9 | | | - Measurement duration for one position | 120 s | | | **Confocal imaging** | | | | | | - Equipment | Zeiss LSM 780, AxioObserver | | | | | - Objective | Plan-Apochromat 40x N.A. 0.95 Korr | | | | | - Excitation wavelength | 488 nm | | | | | - Detection wavelength | 493 – 598 nm | | | | | - Scaling (per Pixel) | 0.42 µm x 0.42 µm x 0.78 µm | | | | | - Image size (Pixels) | 512 x 512 | | | | | - Image size (scaled) | 212.55 µm x 212.55 µm | | | | | - Pixel time | 1.27 µs | | | | | - Frame time | 0.78 s | | | | | - Interval between two cycles | 10 min | | | | | - Number of positions for each cycle | 36 positions | | | | | - Z-stack at each position | 12 slices  (8.6 µm) | 14 slices  (10.2 µm) | 14 slices  (10.2 µm) | 16 slices  (11.8 µm) | | - Total imaging period | 8 h 20 min | 10 h 20 min | 10 h 20 min | 12 h 50 min | | - Total number of imaging cycles | 47 cycles | 60 cycles | 62 cycles | 73 cycles | | - Total number of images | 20,304 | 30,240 | 31,248 | 42,048 | | - Size of data set | 9.94 GB | 14.80 GB | 15.20 GB | 21.01 GB | |  |
| --- | --- | --- | --- | --- | --- | --- | --- | --- | --- | --- | --- | --- | --- | --- | --- | --- | --- | --- | --- | --- | --- | --- | --- | --- | --- | --- | --- | --- | --- | --- | --- | --- | --- | --- | --- | --- | --- | --- | --- | --- | --- | --- | --- | --- | --- | --- | --- | --- | --- | --- | --- | --- | --- | --- | --- | --- | --- | --- | --- | --- | --- | --- | --- | --- | --- | --- | --- | --- | --- | --- | --- | --- | --- | --- | --- | --- | --- | --- | --- | --- | --- | --- | --- | --- | --- | --- | --- | --- | --- | --- | --- | --- | --- | --- | --- | --- | --- | --- | --- | --- | --- | --- | --- | --- | --- | --- | --- | --- | --- | --- | --- | --- | --- | --- | --- | --- | --- | --- | --- | --- | --- | --- | --- | --- | --- | --- | --- | --- | --- | --- | --- | --- | --- | --- | --- | --- | --- | --- | --- | --- | --- |
|  | |

Supplementary Table 2 – Formula of M9 medium supplemented with casamino acids[^3^](#_ENREF_3)

| **No** | **Chemical name** | **Chemical formula** | **Brand** | **M_w_**  **(g/mol)** | **Working concentration** | **Mass at working concentration (g/L)** |
| --- | --- | --- | --- | --- | --- | --- |
| 1 | Calcium chloride dihydrate | CaCl_2_.2H_2_O | Merck | 147.01 | 0.1 mM | 0.01 |
| 2 | Magnesium sulfate heptahydrate | MgSO_4_.7H_2_O | Merck | 246.48 | 2.0 mM | 0.49 |
| 3 | M9 salt |  |  |  |  |  |
| (a) | Sodium phosphate dibasic | Na_2_HPO_4_ | Fisher | 177.99 | 48.0 mM | 8.54 |
| (b) | Potassium dihydrogen phosphate | KH_2_PO_4_ | Merck | 136.09 | 22.0 mM | 2.99 |
| (c) | Sodium chloride | NaCl | Merck | 58.44 | 9.0 mM | 0.53 |
| (d) | Ammonium chloride | NH_4_Cl | Merck | 53.49 | 19.0 mM | 1.02 |
| 4 | Glucose | D(+)-Glucose | BDH | 180.16 | 0.04% w/v | 0.40 |
| 5 | Casamino acids | - | BD Bacto | - | 0.2% w/v | 2.00 |

Supplementary Table 3 – Conversion of actual experiment time and imaging cycle number

| **Actual experiment time  *t* (min)** | **Imaging cycle *n*** | | | |
| --- | --- | --- | --- | --- |
|  | **0.1 ml h^-1^**  **(Run 1)** | **0.5 ml h^-1^**  **(Run 1)** | **1.5 ml h^-1^**  **(Run 1)** | **4.0 ml h^-1^**  **(Run 1)** |
| 10 | 1 (*n_0_*) | 1 (*n_0_*) | 1 (*n_0_*) | 1 (*n_0_*) |
| 20 | 2 | 2 | 2 | 2 |
| 30 | 3 | 3 | Missed | Missed |
| 40 | Missed | 4 | 3 | 3 |
| 50 | Missed | 5 | 4 | 4 |
| 60 | 4 | 6 | 5 | 5 |
| 70 | 5 | 7 | 6 | 6 |
| 80 | 6 | 8 | 7 | 7 |
| 90 | 7 | 9 | 8 | 8 |
| 100 | 8 | 10 | 9 | 9 |
| 110 | 9 | 11 | Missed | 10 |
| 120 | 10 (*n_a_*) | 12 (*n_a_*) | 10 (*n_a_*) | 11 (*n_a_*) |
| 130 | 11 | 13 | 11 | 12 |
| 140 | 12 | 14 | 12 | 13 |
| 150 | 13 | 15 | 13 | 14 |
| 160 | 14 | 16 | 14 | 15 |
| 170 | 15 | 17 | 15 | 16 |
| 180 | 16 | 18 | 16 | 17 |
| 190 | Missed | 19 | 17 | 18 |
| 200 | 17 | 20 | 18 | 19 |
| 210 | 18 | 21 | 19 | 20 |
| 220 | 19 | 22 | 20 | 21 |
| 230 | 20 | 23 | 21 | 22 |
| 240 | 21 | 24 | 22 | 23 |
| 250 | 22 | 25 | 23 | 24 |
| 260 | 23 | 26 | 24 | 25 |
| 270 | 24 | 27 | 25 | 26 |
| 280 | 25 | 28 | 26 | 27 |
| 290 | 26 | 29 | 27 | 28 |
| 300 | 27 | 30 | 28 | 29 |
| 310 | 28 | 31 | 29 | 30 |
| 320 | 29 | 32 | 30 | 31 |
| 330 | 30 | 33 | 31 | 32 |
| 340 | 31 | 34 | 32 | 33 |
| 350 | 32 | 35 | 33 | 34 |
| 360 | 33 | 36 | 34 | Missed |
| 370 | 34 | 37 | 35 | Missed |
| 380 | 35 | 38 | 36 | Missed |
| 390 | 36 | 39 | 37 | 35 |
| 400 | 37 | 40 | 38 | 36 |
| 410 | 38 | 41 | 39 | 37 |
| 420 | 39 | 42 | 40 | 38 |
| 430 | 40 | 43 | 41 | 39 |
| 440 | 41 | 44 | 42 | 40 |
| 450 | 42 | 45 | 43 | 41 |
| 460 | 43 | 46 | 44 | 42 |
| 470 | 44 | 47 | 45 | 43 |
| 480 | 45 | 48 | 46 | 44 |
| 490 | 46 | 49 | 47 | 45 |
| 500 | 47 | 50 | 48 | 46 |
| 510 | - | 51 | 49 | 47 |
| 520 | - | 52 | 50 | 48 |
| 530 | - | 53 | 51 | 49 |
| 540 | - | 54 | 52 | 50 |
| 550 | - | 55 | 53 | 51 |
| 560 | - | 56 | 54 | 52 |
| 570 | - | 57 | 55 | 53 |
| 580 | - | 58 | 56 | 54 |
| 590 | - | 59 | 57 | 55 |
| 600 | - | 60 | 58 | 56 |
| 610 | - | 61 | 59 | 57 |
| 620 | - | 62 | 60 | 58 |
| 630 | - | - | - | 59 |
| 640 | - | - | - | 60 |
| 650 | - | - | - | 61 |
| 660 | - | - | - | 62 |
| 670 | - | - | - | 63 |
| 680 | - | - | - | 64 |
| 690 | - | - | - | 65 |
| 700 | - | - | - | 66 |
| 710 | - | - | - | 67 |
| 720 | - | - | - | 68 |
| 730 | - | - | - | 69 |
| 740 | - | - | - | 70 |
| 750 | - | - | - | 71 |
| 760 | - | - | - | 72 |
| 770 | - | - | - | 73 |

| Supplementary Video 1 | Confocal images of biofilm development at 36 positions at flow rate 0.1 ml h^-1^  The complete biofilm development behavior from bacteria attachment, microcolony formation and maturation to dispersal at flow rate Q = 0.1 ml h^-1^ per inlet over a period of 8 h 20 min. Each frame in the video is a collage of 36 images, each of which is the maximum intensity projection of the Z-stack (12 slices with 0.78 µm interval) at the respective position. |
| --- | --- |
| Supplementary Video 2 | Confocal images of biofilm development at 36 positions at flow rate 0.5 ml h^-1^  The complete biofilm development behavior from bacteria attachment, microcolony formation and maturation to dispersal at flow rate Q = 0.5 ml h^-1^ per inlet over a period of 10 h 20 min. Each frame in the video is a collage of 36 images, each of which is the maximum intensity projection of the Z-stack (14 slices with 0.78 µm interval) at the respective position. |
| Supplementary Video 3 | Confocal images of biofilm development at 36 positions at flow rate 1.5 ml h^-1^  The complete biofilm development behavior from bacteria attachment, microcolony formation and maturation to dispersal at flow rate Q = 1.5 ml h^-1^ per inlet over a period of 10 h 20 min. Each frame in the video is a collage of 36 images, each of which is the maximum intensity projection of the Z-stack (14 slices with 0.78 µm interval) at the respective position. |
| Supplementary Video 4 | Confocal images of biofilm development at 36 positions at flow rate 4.0 ml h^-1^  The complete biofilm development behavior from bacteria attachment, microcolony formation and maturation to dispersal at flow rate Q = 4.0 ml h^-1^ per inlet over a period of 12 h 50 min. Each frame in the video is a collage of 36 images, each of which is the maximum intensity projection of the Z-stack (16 slices with 0.78 µm interval) at the respective position. |
| Supplementary Video 5 | Normalized total biovolume at 36 positions over time at 4 flow rates  Normalized total biovolume per imaging window, *Vnorm_pn_*, of the biofilm over the full experiment duration at 36 positions at four different flow rates (0.1, 0.5, 1.5 and 4.0 ml h^-1^ – Run 1). For each flow rate, all data were normalized against the total biovolume at the respective positions at 2-h time point. Note that the scale for *y-*axis at flow rate 4.0 ml h ^-1^ from 7 h 50 min onwards is different from the rest. |
| Supplementary Video 6 | Total biovolume at 36 positions over time at 4 flow rates  Un-normalized data of the total biovolume per imaging window, *V_pn_*, of the biofilm at 36 positions at four different flow rates (0.1, 0.5, 1.5 and 4.0 ml h^-1^ – Run 1). Note that the scale for *y-*axis at flow rate 4.0 ml h ^-1^ from 7 h 50 min onwards is different from the rest. |
| Supplementary Video 7 | Confocal images of biofilm development at position 7a at 4 flow rates  The video shows biofilm development over the full experiment duration at four different flow rates (0.1, 0.5, 1.5 and 4.0 ml h^-1^ – Run 1) at position 7a. |
| Supplementary Video 8a | Cluster distribution at position 7a over time at 4 flow rates  Cluster size,*V_pni_*, distribution over the entire experiment duration at four different flow rates (0.1, 0.5, 1.5 and 4.0 ml h^-1^ – Run 1) at position 7a. *N_pn_* is the total number of cluster present in the imaging window. The increased in height of the distribution indicates growth while the spreading of the distribution to the right indicates dispersal. Note that the scale for *y-*axis at flow rate 4.0 ml h ^-1^ from 7 h 50 min onwards is different from the rest. |
| Supplementary Video 8b | Theoretical cluster distribution vs experimental cluster distribution at position 7a over time at 4 flow rates  This video compares the calculated (grey) and experimental (black) cluster distribution at position 7a at 4 flow rates, from the start of experiment until the time at maximal growth. Note that the scale for *y-*axis at flow rate 4.0 ml h ^-1^ from 7 h 10 min onwards is different from the rest. Details of calculation of theoretical cluster distribution can be found in Supplementary Note 3. |
| Supplementary Video 9 | Bubble plot at position 7a over time at 4 flow rates  The video shows the spatial distribution of individual cluster size, *V_pni_*, at four flow rates (0.1, 0.5, 1.5 and 4.0 ml h^-1^ – Run 1) at position 7a. The circles, representing the clusters, were plotted at the centroid of the respective clusters with their area representing the magnitude of their respective *V_pni_*. |
| Supplementary Video 10 | Total biovolume vs time for 36 positions at 4 flow rates  The video shows the total biovolume in the imaging window, *V_pn_*, vs. time, *t*, from *t* = 0 to the end of each experiment for all 36 positions at four flow rates (0.1, 0.5, 1.5 and 4.0 ml h^-1^ – Run 1). |
| Supplementary Video 11 | Biofilm formation and sloughing under high flow rate 4.0 ml h^-1^  The video shows biofilm formation and sloughing at position 7b under high flow rate 4.0 ml h^-1^ (Run 2). Quantitative analysis was not conducted on this run due to low number of attached clusters after inoculation ($\overline{N_{a}}$ < 10). |

List of variables

| Variables | Definition |
| --- | --- |
| *x* | *x* coordinate |
| Q | flow rate per inlet |
| *p* | position number |
| *n* | imaging cycle number |
| *n_0_* | imaging cycle at the start of experiment |
| *n_a_* | imaging cycle at 2 h into the experiment |
| *n_pmax_* | imaging cycle of maximal growth at position *p* |
| *t* | actual experiment time |
| *V_pni_* | biovolume of individual cluster *i* at position *p* and imaging cycle *n* |
| *V_pnimax_* | maximum size of cluster in all positions before initiation of dispersal |
| *V_pn_* | total biovolume per imaging window at position *p* and imaging cycle *n* |
| *V_pnmax_* | maximum *V_pn_* at position *p* before initiation of dispersal |
| $\overline{V_{pn_{\max}}}$ | average *V_pnmax_* over three adjacent positions a, b and c |
| *Vnorm_pn_* | *V_pn_* normalized against *V_pna_* |
| *N_pn_* | total number of clusters at position *p* and imaging cycle *n* |
| $N_{pn_{a}}$ | total number of clusters attached at time 2 h at position *p* (i.e. total number of clusters at position *p* and imaging cycle *n_a_)* |
| $\overline{N_{a}}$ | average of $N_{pn_{a}}$over three adjacent positions a, b and c |
| *g_p_* | apparent growth rate at position *p* at time *t* |
| $\overline{g_{p}}$ | average growth rate at position *p* |
| *t_dp_* | doubling time at position *p* |
| $\overline{t_{d}}$ | average doubling time over three adjacent positions a, b and c |
| *D_pni_* | diameter of bubble representing individual cluster *i* at position *p* and imaging cycle *n* |
| *t_pdisp_* | time at the initiation of dispersal at position *p* |
| $\overline{t_{disp}}$ | average of *t_pdisp_* over three adjacent positions a, b and c |
|  | average of dispersal time at positions 12 a, b and c |
| $\Delta\overline{t_{disp}}$ | time difference between the initiation of dispersal at position 12 and position 1 |
|  |  |
|  |  |
|  |  |

**REFERENCES**
